# Effects of standardized mindfulness programs on burnout: a systematic review and original analysis from randomized controlled trials

**DOI:** 10.3389/fpubh.2024.1381373

**Published:** 2024-05-22

**Authors:** Dyna Shoker, Laura Desmet, Nelly Ledoux, Anne Héron

**Affiliations:** ^1^Unité de Recherche Clinique ARC EN CIEL UF657-Centre Hospitalier Victor Jousselin-GHT HOPE Les Hôpitaux Publics Euréliens, Dreux, France; ^2^Cabinet médical de la gare de Nyon, Nyon, Switzerland; ^3^Faculté de Santé - Pharmacie, Université Paris Cité, Paris, France

**Keywords:** mindfulness, burnout, randomized controlled trials, systematic review, work, stress

## Abstract

According to the World Health Organization (WHO), burnout is a syndrome conceptualized as resulting from chronic occupational stress that has not been successfully managed. It is characterized by emotional exhaustion, cynicism toward work, and a lack of personal accomplishment at work. Recent WHO guidelines on mental health suggest that mindfulness could have beneficial effects in a professional environment, but to the best of our knowledge, there is currently no study that has made a large inventory of research focused specifically on the effects of standardized programs on burnout. Which professional populations have already been studied? What are the characteristics of the programs? Have studies shown a significant effect and on what indicator?

**Objective:**

To assess the effects of standardized programs of mindfulness on burnout, we carried out a systematic review using an exhaustive inventory of the international literature based on randomized controlled trials (RCTs).

**Methods:**

The articles were selected according to PRISMA recommendations. The Embase, PubMed/MEDLINE, EBSCOhost, HAL databases were searched with the keywords “mindfulness,” “burnout,” and “randomized” in the title and abstract of each article. The data were all collected in an Excel spreadsheet and analyzed in pivot tables, which were then presented in graphs and maps.

**Results:**

A total of 49 RCTs were thus selected, the majority of which were of good methodological quality, of American origin (43% of studies), concerned professionals in the health sector (64% of participants included), and mostly women (76%). The RCTs assessed the effects of 31 different mindfulness programs, mostly with the Maslach Burnout Inventory (78% of RCTs). More than two-thirds of RCTs (67%) showed a significant beneficial effect on burnout measurement indicators, with emotional exhaustion being the most impacted component.

**Conclusion:**

This systematic review shows that mindfulness-based interventions could be approaches of choice to prevent emotional distress of burnout. Further studies are still needed to determine which type of program is best suited to impact the two other components of burnout.

## 1 Background

### 1.1 Burnout

According to the World Health Organization (WHO), burnout is a syndrome conceptualized as resulting from chronic workplace stress that has not been successfully managed. It has three dimensions: feelings of energy depletion or exhaustion; increased mental distance from one’s job, or feelings of negativism or cynicism related to one’s job; and reduced professional efficacy (1).

The absence of a consensual, unequivocal definition, its multifactorial origin, vagueness, and subjectivity in diagnostic criteria complicates the identification and prevalence of burnout in a general population (2). Estimates report values as high as 20% of the working population, but the number is highly dependent on threshold values for defining burnout (3). During the COVID-19 pandemic, healthcare professionals had to manage the increased workload, shortage of personal protective equipment, emotional burden, the anxiety around this new disease, and the stigmatization of caregivers as potential vectors of viral infection. All these factors have generated stress at work (4). The prevalence of burnout was 52% among all healthcare workers during this pandemic (5).

The social cost of occupational stress is estimated at several billion euros in France, Switzerland, Germany, the Netherlands, and the United States (6). Identifying and preventing burnout syndrome is therefore a current public health issue.

The symptoms of burnout are grouped into four categories (2):

1. Emotional and cognitive manifestations such as anxiety, irritability, sad mood, difficulty concentrating, and memory loss.
2. Behavioral manifestations such as withdrawal and aggressive behavior.
3. Motivational manifestations such as progressive disengagement and decrease in self-esteem or motivation.
4. Physical manifestations such as asthenia, digestive disorders, nausea, and headaches.

There are two types of risk factors: work-related and individual. Work-related factors are determined by high work demands (intensity and working time), high emotional demands, lack of autonomy, poor social and labor relations, poor appreciation of their work values, and insecurity about the work situation (7, 8). Some studies agree on the fact that factors related to the individual such as emotional instability, anxiety, and a history of depression can also influence the occurrence of burnout (9, 10).

Burnout can be detected by a general practitioner through a history and clinical examination, looking for work-related risk factors, individual factors, and clinical manifestations of burnout (11). To help identify burnout, measurement tools have been developed. These are self-administered questionnaires. Combined with an interview and a clinical examination, these self-questionnaires can guide an interview but should not be used as a diagnostic tool alone (11, 12). These assessment tools are therefore more intended to assess the level of burnout based on elements that define it. Among them, the Maslach Burnout Inventory (MBI) is the most used. This questionnaire was then diverted from its primary role, and many studies now use it incorrectly as a diagnostic tool. Similarly, other questionnaires such as the Oldenburg Burnout Inventory(OLBI), the Professional Quality Of Life (proQOL), the Shirom Melamed Burnout Questionnaire (or Measure), and the Copenhagen Burnout Inventory have emerged (13–20).

Treatment consists of treating burnout as well as acting on the professional risk factors at the origin of the disorder. A temporary break from work is most often prescribed. The general practitioner treats any symptoms based on the diagnostic approach and refers the patient to a psychiatrist if necessary. The latter can carry out a psychopathological diagnosis, rehabilitate the treatment, and take charge of one of the complications of burnout (depression). Support based on psychotherapies or mind–body interventions can be proposed in order to carry out “work on oneself,” before returning to work (21, 22). Post-rehabilitation or preventive actions (individual and/or collective) are recommended to avoid any risk of relapse (22). Mind–body interventions are practices that focus on the interactions among the brain, body, mind, and behavior with the intent of using the mind to alter physical function and promote overall health (23). Mindfulness meditation is considered a mind–body practice. In addition, mindfulness is considered a “Third Wave” Cognitive and Behavioral Therapy, which gives an important place to emotions and their bodily components, as well as to the meaning that the individual wishes to give to his life (24).

### 1.2 Mindfulness

The word “mindfulness” refers to a notion derived from traditional meditation practices meaning “wise attention” (25). It can be defined as awareness that arises through paying attention, on purpose, in the present moment, non-judgmentally and with acceptance (26, 27). Mindfulness was introduced into the Western scientific world by Jon Kabat-Zinn through the mindfulness-based stress reduction (MBSR) program at the Faculty of Medicine of the University of Massachusetts, in the form of a standardized, secular educational format to relieve the stress of healthcare professionals and the suffering of chronic pain patients (27, 28). Over the past 50 years, MBSR became the “gold standard,” many other derivative mindfulness-based protocols have been developed around the world and are the subject of intense research (29–31). The training is either provided face-to-face or online digital format, followed by the use of pre-recorded audio support by an instructor for daily practices (32, 33). Mindfulness practice and programs, often referred to as mindfulness-based interventions, have become increasingly popular in every sector of society, including healthcare, education, business, and government (31).

Mindfulness develops through formal meditation practices (sitting, lying down, or moving) as well as informal practices of attentive presence in daily activities such as brushing teeth and taking a shower.

These practices are essentially based on two types of meditation that contribute to the development of attentional capacities and emotional regulation (34, 35).

- Focused attention: the person’s attention is directed and maintained voluntarily and in a sustained manner on a chosen object. It can be both material objects perceived by the five senses such as sounds and bodily sensations and mental objects such as the current emotional state. This practice helps to develop attention skills, which gradually improves focus and promotes mental stability (36–39).
- Open monitoring (choiceless awareness) involves being present to the experience, without seeking to control it, fully welcoming the mental processes that arise in awareness. It is thus possible to become aware of one’s own automatisms, such as mental habits, emotional reactions, or judgments, which generally operate unconsciously. Open presence meditation develops metacognitive abilities as well as emotional flexibility (28, 37, 40–42).

Other closely related meditation practices can, in some cases, be associated with the two previous ones. These are the meditations of loving kindness or compassion. These practices cultivate the wish for happiness or the absence of suffering for oneself, one’s loved ones, and all other beings. It promotes the deployment of the prosocial qualities of altruism, generosity, love, compassion, and tolerance (34, 43, 44).

In parallel with the neurocognitive effect of meditation practices on attention control, emotion regulation, self-awareness, and prosocial skills, studies have shown beneficial changes in brain activity and, in the longer term, in neuroplasticity (45–50).

Meta-analyses suggest beneficial effects of mindfulness-based interventions on sleep quality (51) and mental health, particularly in anxiety disorders and relapse of depression (52–55).

The recent World Health Organization *guidelines on Mental health at work* provide that psychosocial interventions that aim to build workers’ skills in stress management—such as approaches based on mindfulness—may promote positive mental health, reduce emotional distress, and improve work effectiveness (56). Studies carried out with health professionals also showed more empathy, better communication between professionals, better emotional regulation and increase in well-being (57–60). Additionally, recent meta-analysis showed that mindfulness might have beneficial effects on burnout among health workers, but few RCTs (2–6) were included in these studies (61–66).

Despite this array of arguments showing that mindfulness has beneficial effects in professional environment, to the best of our knowledge, there is currently no systematic study that has made a large inventory of the international research focused specifically on burnout. Which professional populations have already been studied? What are the characteristics of the mindfulness programs that have been tested? Have studies shown a significant effect and on what indicator of burnout?

Here, we carried out a systematic review to assess the effects of standardized programs of mindfulness on burnout, using an exhaustive inventory of the international literature on this topic. We collected and analyzed the available data from all the randomized controlled trials, conducted between 2006 and 2022, which are considered to have a very high level of evidence in intervention research.

## 2 Methods

This systematic review was conducted according to the Preferred Reporting Items for Systematic Reviews and Meta-Analyses (PRISMA) guidelines (67). The protocol was registered in PROSPERO (Registration 2022: CRD42022383475). The selection of articles and the data analysis were performed by four investigators. The data collected were analyzed, synthesized, and presented as can be when following the Evidence & Gap Maps methodology, thus providing a visual presentation of the results in a user-friendly format (68).

### 2.1 Eligibility criteria

Articles were selected by two researchers independently (D.S. and L.D.), based on the following inclusion criteria: (1) randomized controlled trials (RCTs) published in peer-reviewed scientific journals; (2) the experimental group that only received a reproducible mindfulness training; (3) burnout assessment; (4) article written in English or French; and (5) full-text accessible.

Studies that did not meet the eligibility criteria were excluded. This was the case for articles that combined mindfulness intervention with other training such as fitness, relaxation, psychoeducation, and art therapy.

### 2.2 Information sources and search strategy

The search was carried out by querying the following three international databases of scientific articles: PubMed/MEDLINE, Embase, EBSCOhost, as well as the French database HAL. These databases were consulted between October 26 and October 28, 2022 (inclusive). The keywords selected for the search strategy were: “mindfulness,” “burnout,” and “randomized.” They were searched in the title and/or abstract of each article. The full search strategies for each database are available in Supplementary Table S1.

### 2.3 Selection process

A first selection was made from reading the title and the abstract. The full texts of the publications were obtained from *Les Bibliothèques d’Université Paris Cité*, or thanks to the generosity of some authors. The selection criteria were applied again when reading these full-text articles.

The data from the different articles were collected in an Excel table by a pair of independent researchers (D.S., L.D., or N.L.). In the event of a discrepancy between the two researchers, a third researcher (A.H) was consulted to reach a consensus.

### 2.4 Data collection

For each randomized controlled trial (RCT), the following data were collected in a spreadsheet: author’s names, year of publication, countries where the studies were conducted, participant’s characteristics (age, gender, and professional sector), sample size, characteristics of mindfulness training (name, duration, type of mindfulness practices, intervention conditions and material, and number of instructors), type of control group (active or passive), burnout scales used, effects, scores and *p*-values on each indicator of burnout after intervention and follow-up, dropout rate, side-effects of mindfulness training, and the quality of RCTs.

### 2.5 Effects on burnout

The effect of mindfulness training on burnout was considered significant when at least one of the indicators of burnout (emotional exhaustion, depersonalization, or personal accomplishment) was significantly impacted by the intervention in comparison with the control groups (*p* ≤ 0.05).

### 2.6 Quality assessment

The methodological quality of the studies included in the review was performed by two researchers (D.S. and L.D.) using the part 2 of the mixed methods appraisal tool (MMAT) (69), which is dedicated specifically to randomized controlled trials. Any disagreements were settled by a third party (A.H.).

This tool was chosen because of its ease of use: it allows a rapid rating of the methodological quality of the studies in five criteria: (1) If the randomization was appropriately performed? (2) Are the groups comparable at baseline? (3) Is there a complete data on outcome? (4) Are outcome assessors blinded to the interventions provided? (5) Did the participants adhere to the assigned intervention? Each item was scored with a Yes, No, or Cannot tell.

For item 3, the authors agreed to consider that the data were complete when the dropout rate was less than 20%.

### 2.7 Analysis and synthesis of the data

After reviewing the included articles, meta-analysis was considered not relevant due to the heterogeneity in the mindfulness trainings (content, duration, training material), participant characteristics, and burnout scales.

A spreadsheet with data analysis was carried out in the form of pivot tables, descriptive statistics, and graphs. The results were synthetized using a graphic analysis and a systematic approach.

## 3 Results

### 3.1 Selection

**Figure 1:**
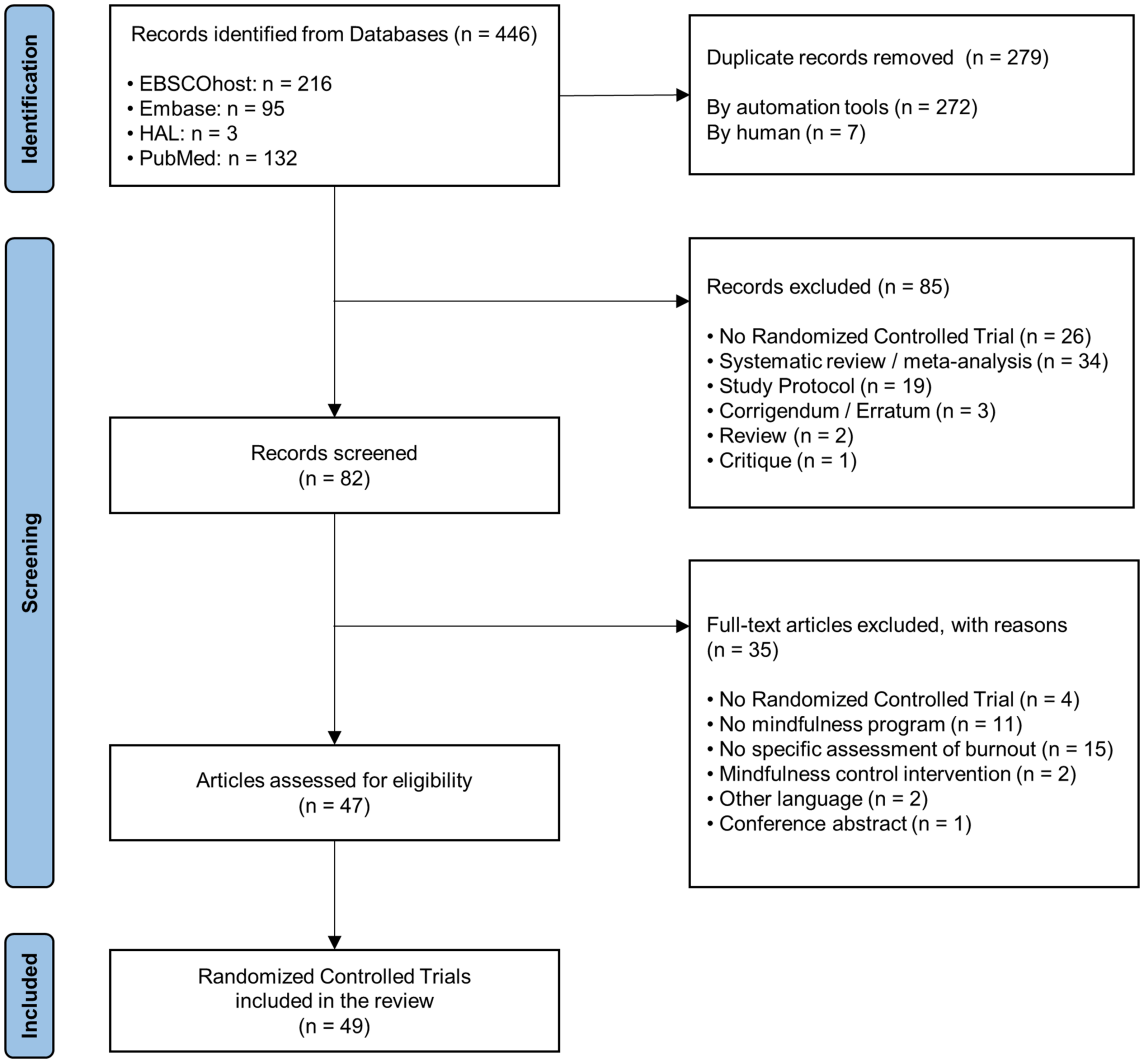
PRISMA flow diagram of the search and study selection process.

The selection of the studies is illustrated in the PRISMA flow diagram of the search and study selection process. Figure 1 using the PRISMA flowchart (67). The search on databases identified 446 articles that were screened for eligibility. After the removal of 279 duplicates and 85 articles deemed ineligible, 82 full-text articles were screened. A total of 47 articles met inclusion criteria. Two of these articles were composed of two studies (different mindfulness interventions or different participants). Finally, 49 RCTs were included in the review. The bibliographic references of each article are available at the end of the Supplementary material.

### 3.2 Study and sample characteristics

Supplementary Table S2 gives the study and sample characteristics for each article.

**Figure 2:**
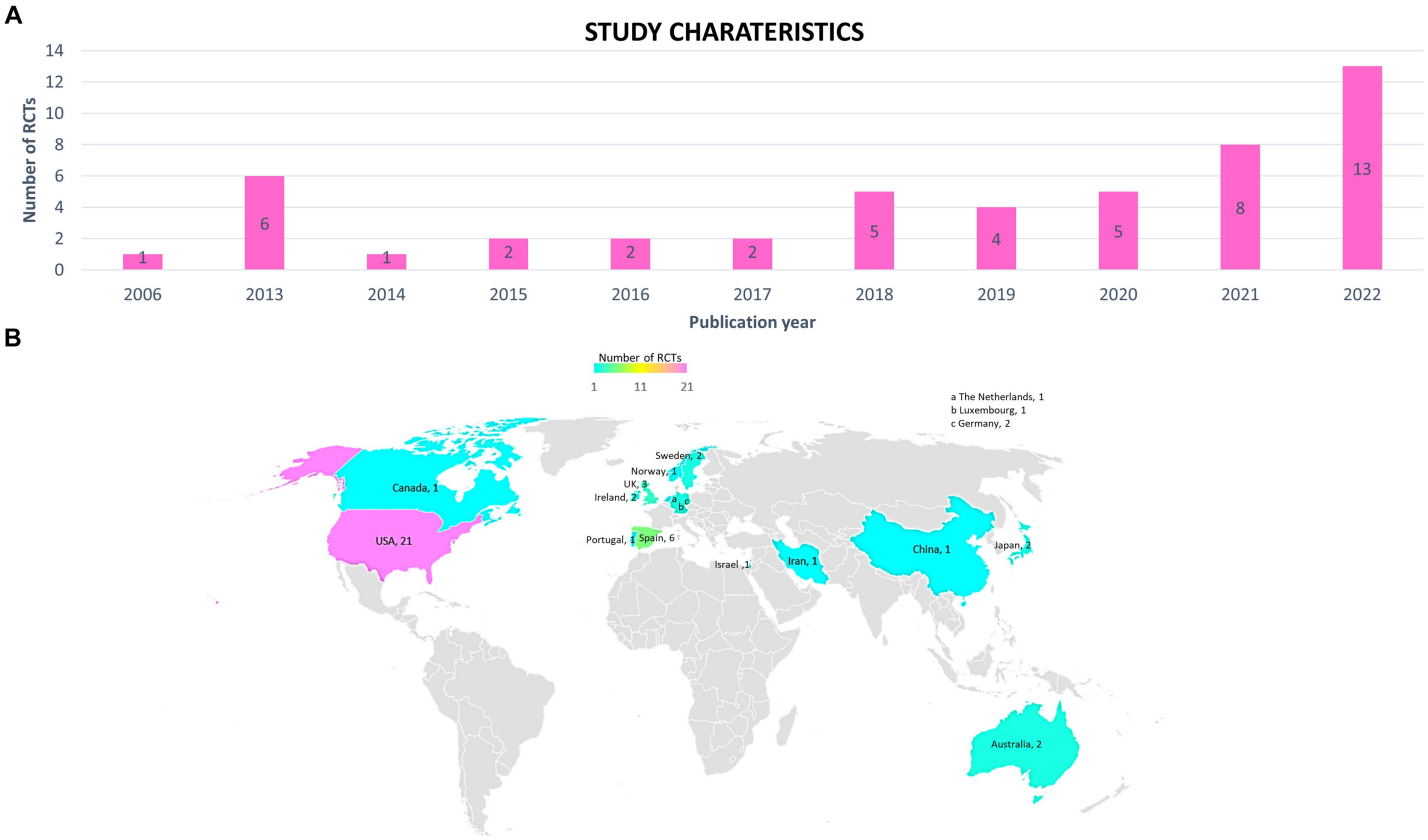
Study characteristics. **(A)** Annual distribution of randomized controlled trials (RCTs). **(B)** Countries where participants were enrolled in the RCTs.

The 49 studies were published between 2006 and 2022. More than half have been published in the last three years (Figure 2A). Studies originated in 16 different countries, but most studies (43%) were conducted in the United States (19 in U.S., one in U.S. and Canada, and one in U.S. and Israel). Nineteen studies enrolled participants in Europe (Germany, Ireland, Luxembourg, Norway, Portugal, Spain, Sweden, The Netherlands, and United Kingdom). Four studies were carried out in Asia and one in Oceania. Three studies did not specify their location (Figure 2B).

**Figure 3:**
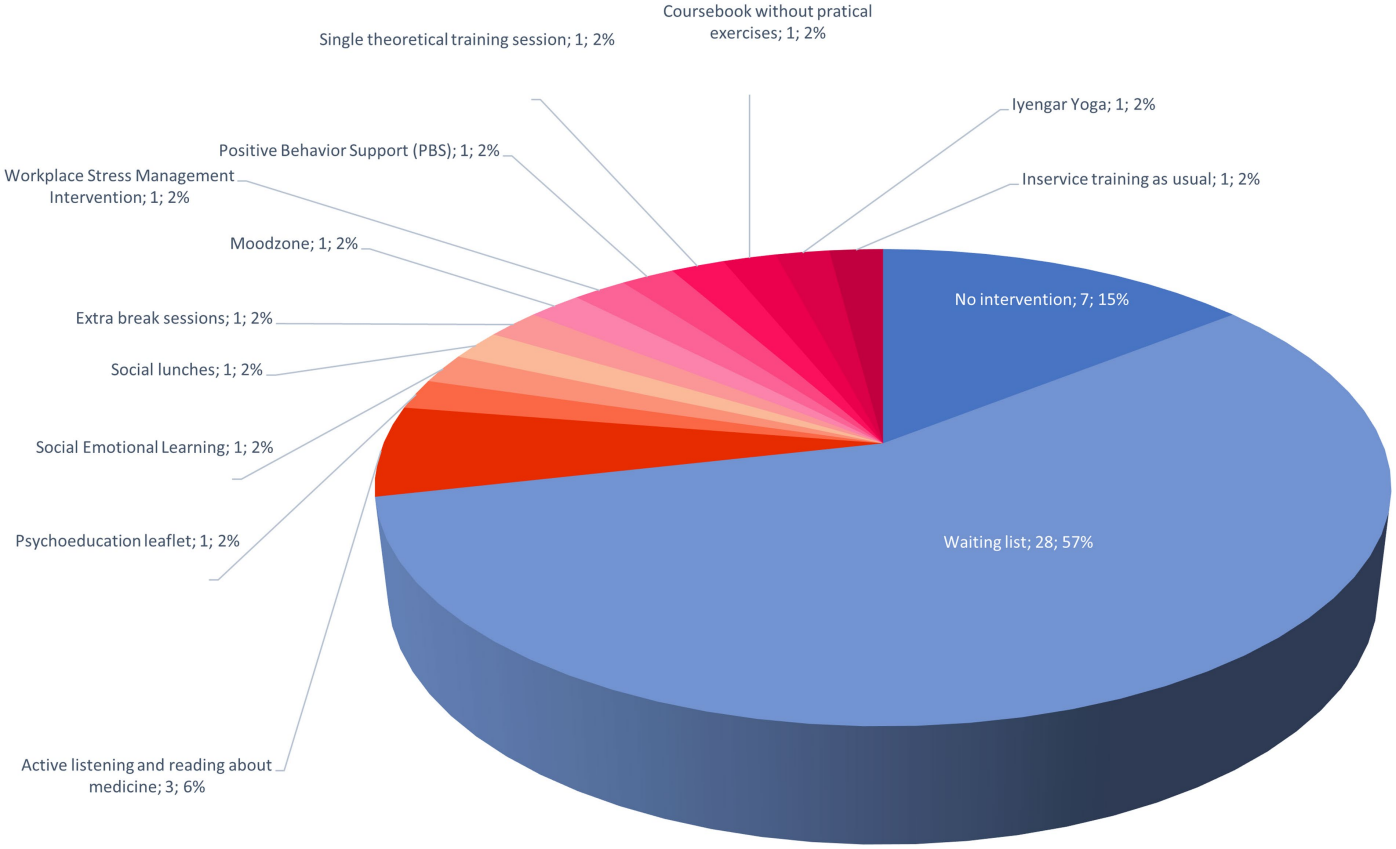
Control groups compared with mindfulness interventions. Passive control groups have been used in 72% of the stuides (blue tones) and active control groups in 28% (red tones).

Of these 49 studies, 35 RCTs compared the mindfulness training with a passive control group (28 “waiting list” and 7 “no intervention”) (Figure 3). The other RCTs included an active control group (active listening and reading about medicine, social emotional learning, social lunches, extra break sessions, Moodzone, workplace stress management and intervention, single theoretical training session, course book without practical exercises, Iyengar Yoga, in-service training as usual, and psychoeducation leaflet).

Depending on the study, the dropout rate of participants ranged from 0 to 63% with an average dropout rate of 17% (Supplementary Table S2).

**Figure 4:**
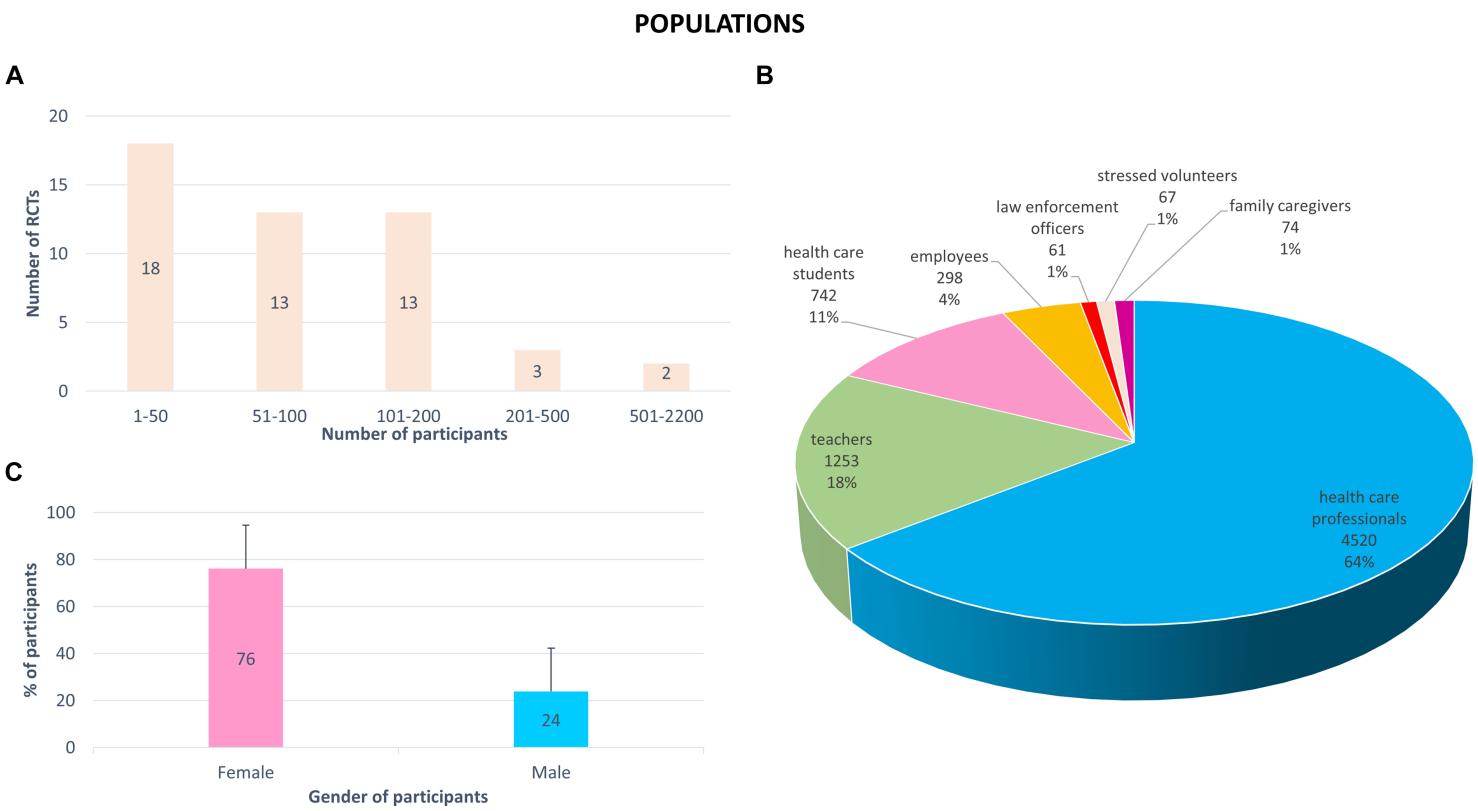
Sociodemographic characteristics of participants included in the 49 randomized controlled trials (RCTs). **(A)** Number of participants included (total: 7015 participants). **(B)** Profession of participants (number of participants and % of total participants includes in all RCTs). **(C)** Percentage of female and male included in all RCTs (mean + standard deviation).

From all the RCTs, a total of 7,015 participants were included. Sample sizes ranged from 16 to 2,182 participants (Supplementary Table S2). The average number of participants included in each study was 143 ± 316 participants (mean ± SD). Thirty-one studies (63%) included fewer than 100 participants (Figure 4A). Subjects were mainly healthcare professionals (29 studies, 64% of the participants included in all RCTs) (Figure 4B). The other participants included were teachers (6 studies, 18%), students (6, 11%), employees (5, 4%), low enforcement officers (1 study, 1%), stressed volunteers (1 study, 1%), and family caregivers (1 study, 1%). Most participants identified as female (76%) (Figure 4C). The average sample age was 39 ± 8 years (mean ± SD). The age range was from 17 to 80 years old (Supplementary Table S2).

### 3.3 Methodological quality of RCTs

The methodological quality of RCTs was assessed using the MMAT (Supplementary Table S3, Supplementary Figure S1). RCTs were mainly judged for good quality: in 78% of the RCTs, the participants received the assigned intervention; 69% of articles mentioned how the randomization schedule was generated; 63% of RCTs presented complete outcome data; for 47% of RCTs, groups were comparable at baseline for sociodemographic characteristics and outcome data; however, the participants were not blinded to assignment in 80% of the studies.

### 3.4 Mindfulness training characteristics

All mindfulness training characteristics are summarized in Supplementary Table S4.

**Figure 5:**
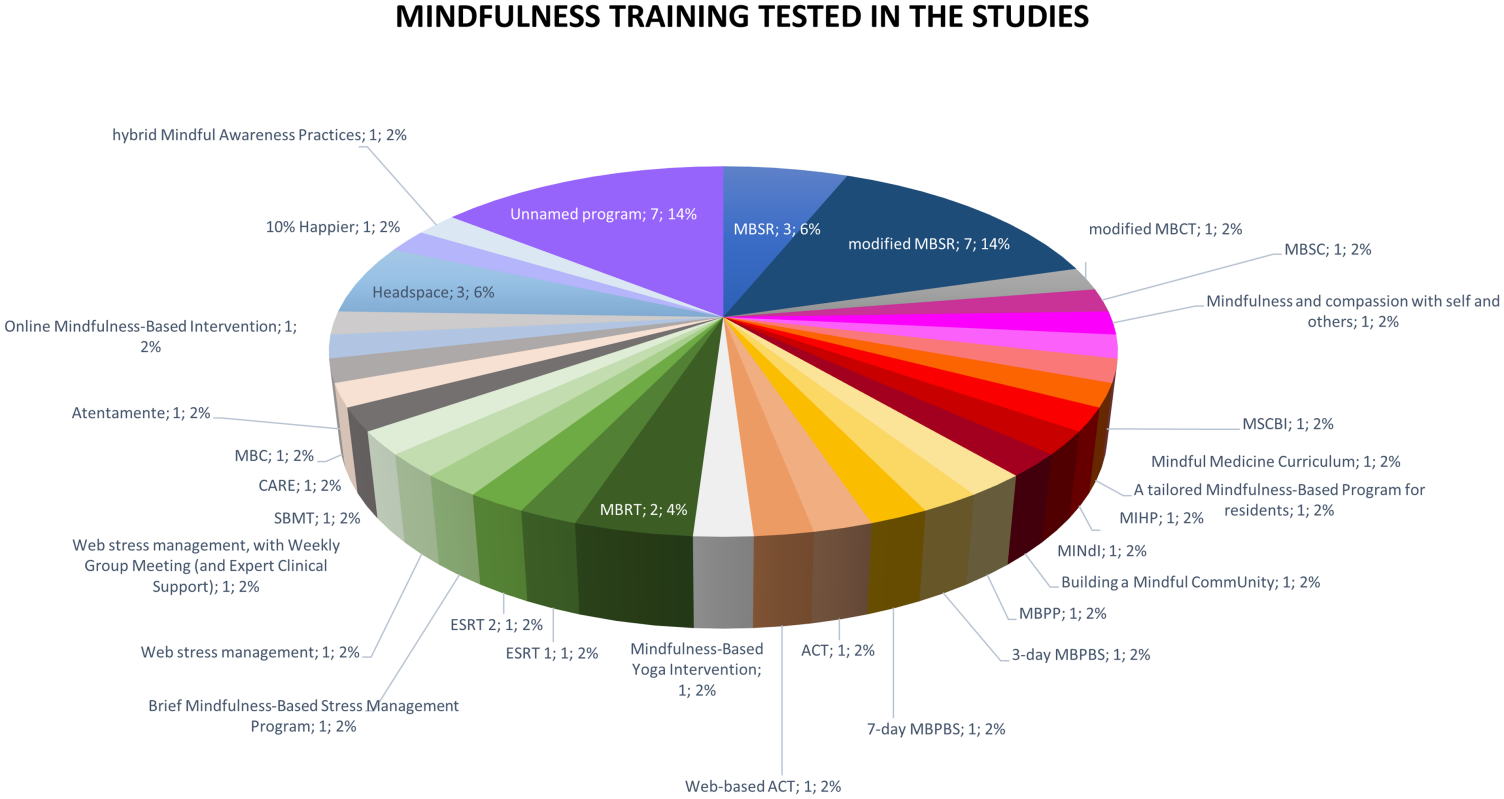
Mindfulness trainings tested in the 49 randomized controlled trials. ACT, acceptance and commitment therapy; CARE, cultivating awareness and resilience in education; ESRT, enhanced stress resilience training; MBC, mindfulness- based course; MBPBS, mindfulness-based positive behavior support; MBPP, mindfulness-based positive psychology; MBRT, mindfulness-based resilience training; MBSC, mindfulness-based self-care; MBSR, mindfulness-based stress reduction; MIHP, mindfulness for interdisciplinary healthcare professionals; MINdI, mindfulness intervention for new interns; MSCBI, mindfulness and self-compassion-based intervention; SBMT, school-based mindfulness training.

Many different mindfulness trainings were tested in the RCTs. A total of 31 different training names were identified. The most studied program was MBSR (mindfulness-based stress reduction), tested in 10 studies (20%), counting modified MBSR (Figure 5). The adaptations of the MBSR program consisted in the modification of duration (condensed or shortened program, from 2 to 26 h), and/or content (omission of movement practices, addition of loving kindness, shortening, or absence of the retreat day).

**Figure 6:**
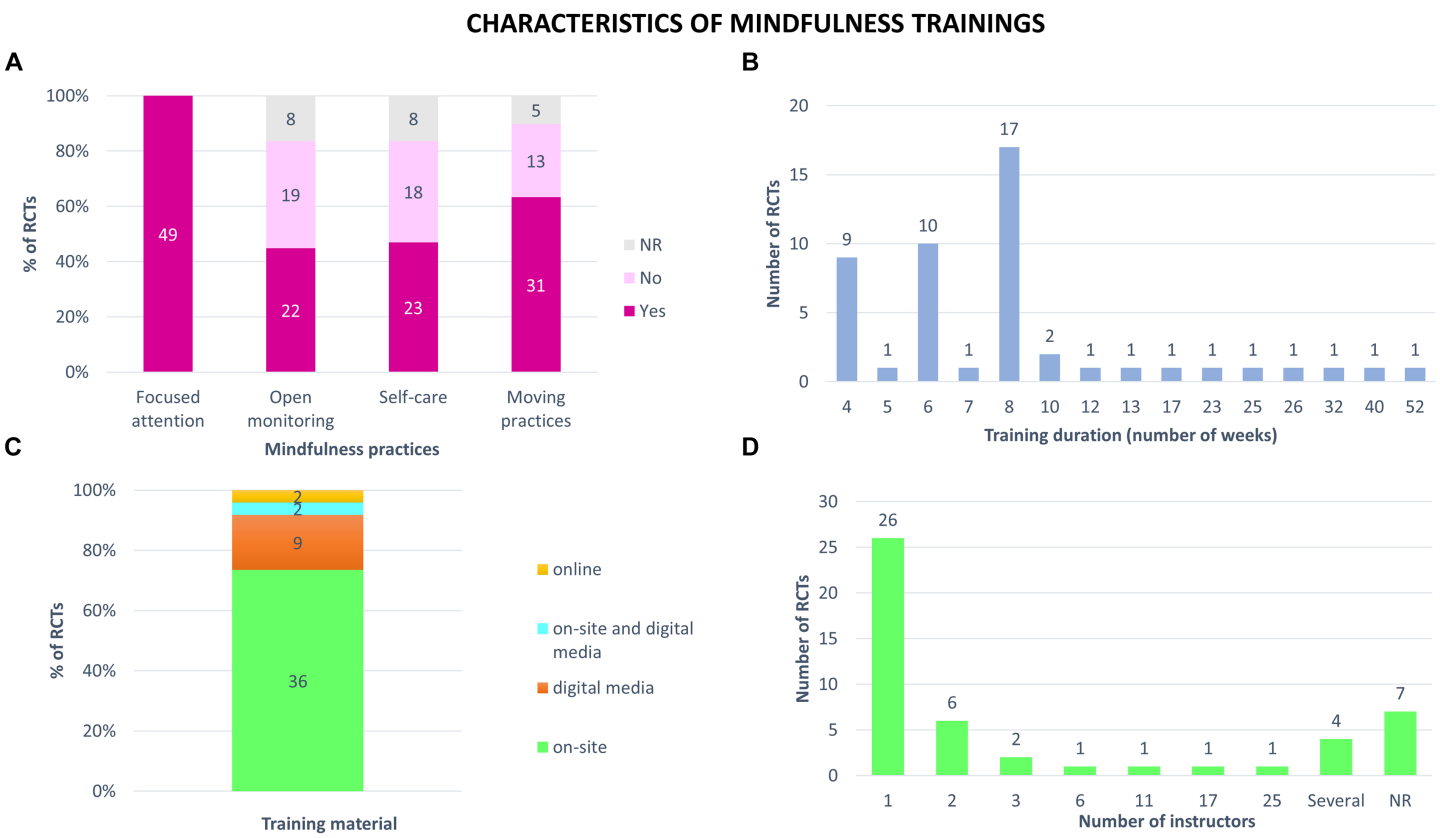
Characteristics of mindfulness trainings tested in the 49 randomized controlled trials (RCTs). **(A)** Types of mindfulness practices identified in each study. **(B)** Duration of trainings proposed. **(C)** Training material used in mindfulness trainings. Trainings took place with an instructor present in person (on-site) or at distance (online); online but only with digital media (digital media); combined face-to-face and distance learning (on-site and digital media). **(D)** Number of instructors who delivered the mindfulness training. Some authors mentioned that there were several instructors but did not give the exact number. NR: not reported.

All the studies included followed “focused attention” as the meditative practice, 22 trainings offered open-monitoring, 23 self-care, and 31 moving practices (Figure 6A).

The training duration ranged from 4 to 52 weeks (Figure 6B). For 35% of the RCTs (n = 17), the trainings lasted 8 weeks. The training hours ranged from 0.8 to 147 h, with an average of 20 ± 25 h (mean ± SD). The average weekly duration of training was 2 ± 1 h, ranging from 0.2 to 4.5 h per week.

About the support of mindfulness teaching, 73% of the studies offered on-site training. For 9 RCTs (18%), all sessions were done at home, using a digital medium (mobile app, recorded sessions, video recording of a face-to-face class). Two RCTs tested trainings a combination face-to-face and distance training. Two interventions included online group sessions (Figure 6C).

More than half of the mindfulness trainings tested involved a single instructor (26 studies). Six interventions were delivered by 2 instructors and 10 by more than 2 instructors. In seven studies, the authors did not specify the number of instructors involved (Figure 6D).

### 3.5 Burnout assessment

Supplementary Table S5 gives main outcomes for each study.

**Figure 7:**
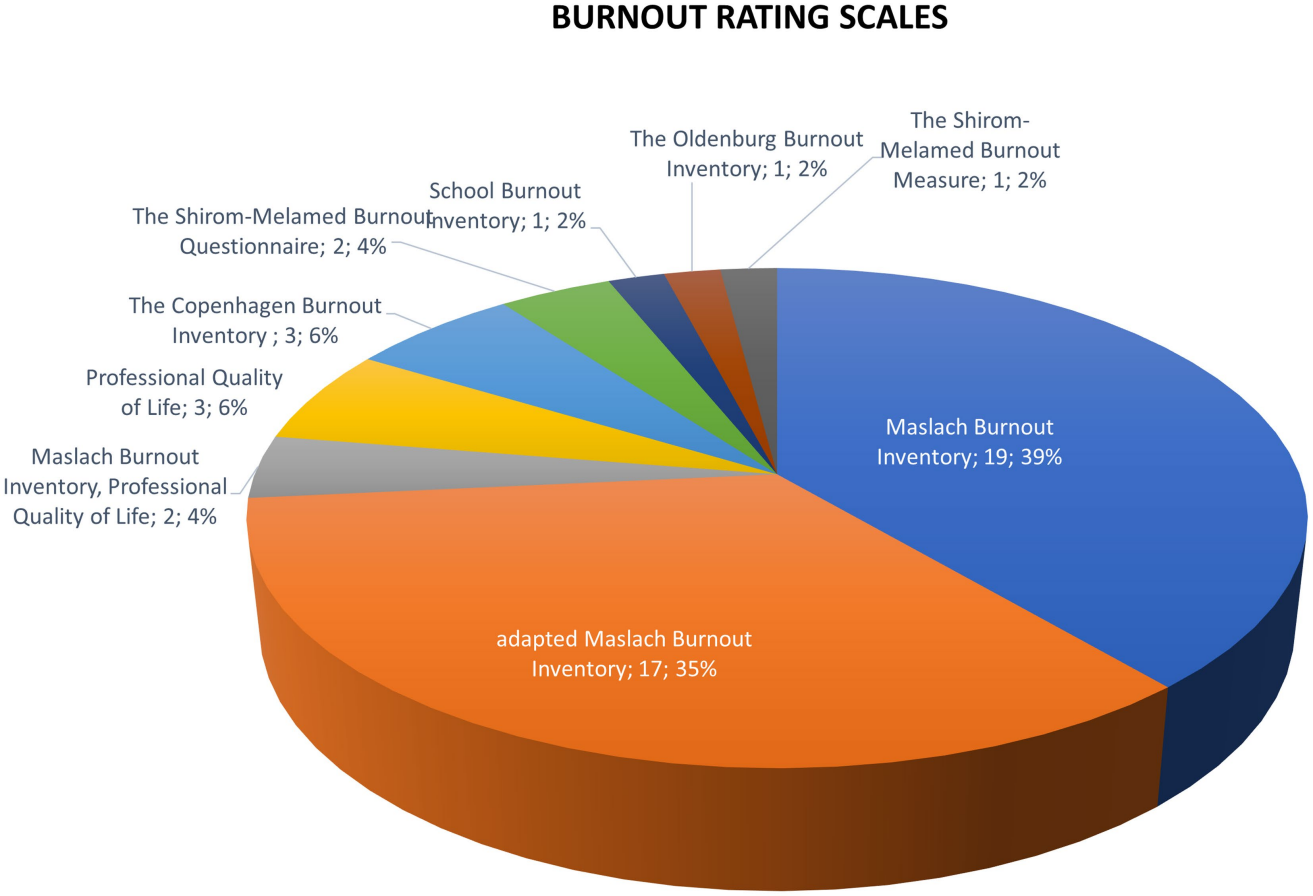
Scales of burnout assessment used in the 49 randomized controlled trials. Adapted Maslach Burnout Inventory scales are modified or abbreviated versions of the original Maslach Burnout Inventory.

In most studies (38, 78%), burnout was measured using the Maslach Burnout Inventory (original version: 19; adapted MBI: 17; associated with the ProQOL: 2) (Figure 7). Number of MBI items used ranged from 2 to 22. Other scales were the Copenhagen Burnout Inventory, the Shirom-Melamed Burnout Questionnaire, the Shirom-Melamed Burnout Measure, the School Burnout Inventory, and the Oldenburg Burnout Inventory.

**Figure 8:**
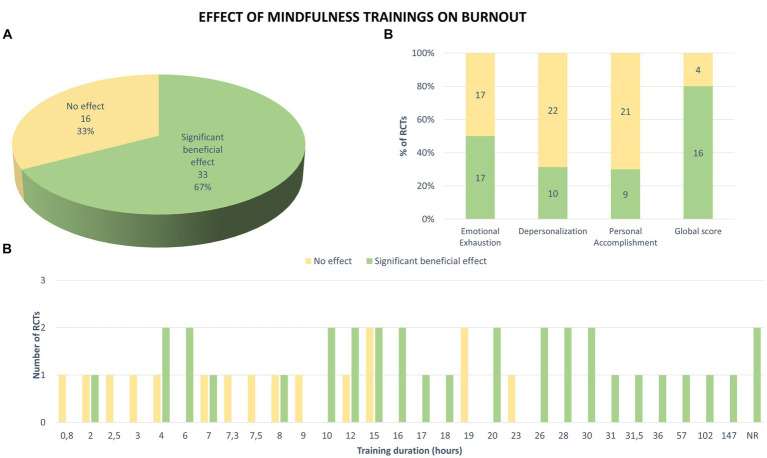
Effects of mindfulness training on burnout measured in the 49 RCTs. **(A)** Significant beneficial effect corresponds to an improvement in at least one indicator of burnout, either after the mindfulness training or at distance from this intervention (follow-up), in comparison with the control group (results correspond to number and percentage of 49 RCTs). **(B)** Mindfulness effects on each dimension of burnout and on global score, measured in the RCTs (numbers on plots indicate number of RCTs with significant effect in green or no effect in yellow). **(C)** Effects of trainings depending on their duration. NR: not reported.

A total of 33 studies (67%) described a significant beneficial effect of mindfulness training on at least one indicator of burnout, either after the intervention or at follow-up (Figure 8A). It must be noted that a total of 10 of the 49 RCTs showed a significant beneficial effect during the follow-up period, while 22 studies (55%) did not compare intervention and control groups at follow-up.

The majority of RCTs (34/49, 69%) evaluated the effect of mindfulness training on emotional exhaustion (Figure 8B). Half of them showed a significant beneficial effect after intervention compared with the control group. For those who assessed depersonalization (32/49, 65%), 31% showed a significant improvement in this aspect. Finally, personal accomplishment was significantly improved in 30% of studies that assessed it (30/49, 61%). Only 20 studies (41%) measured a global score but 80% of them showed a beneficial effect of mindfulness on burnout.

Of the 49 RCTs, 21 involved mindfulness training that lasted at least 16 h (Figure 8C). Among them, 18 studies (86%) showed a significant improvement in burnout compared with the control group. Thus, the more hours or hours per week the program had, the more likely it seemed to have a significant beneficial effect on burnout.

**Figure 9:**
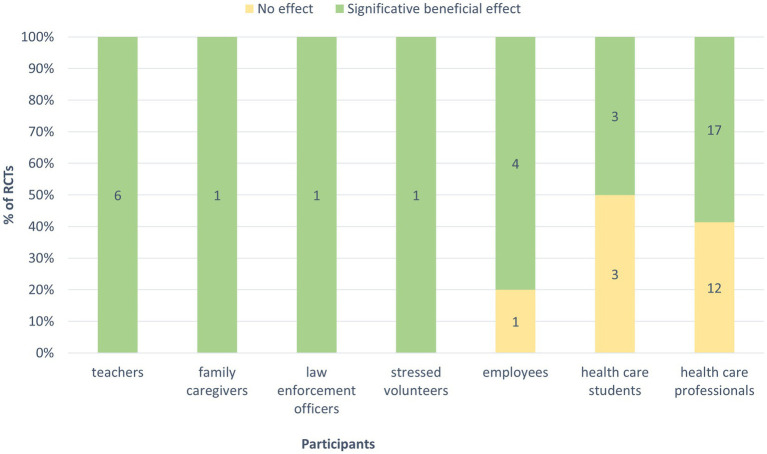
Effects of mindfulness training on burnout according to the participants. Significant beneficial effect corresponds to an improvement in at least one indicator of burnout, either after the mindfulness training or at distance from the intervention (follow-up) in comparison with the control group (numbers on plots indicate number of RCTs with significant effect in green or no effect in yellow).

All six RCTs conducted among teachers showed beneficial effects of mindfulness on burnout (Figure 9). Each of the studies conducted with stressed volunteers, family caregivers and law enforcement officers were effective too. Among employees, only one out of five studies showed no effect of mindfulness training which involved a training delivered only via app. In the population of healthcare students, only three out of six RCTs showed an effect on burnout. Among healthcare professionals, 17 of 29 studies (59%) show a significant beneficial effect (of the 12 that showed no effect, 10 (83%) tested training of less than 16 h duration).

### 3.6 Side effects

Of the 49 studies, 37 (76%) did not address the topic of potential side effects. Nine RCTs specified that no side effects or adverse events were reported by participants. The remaining studies mentioned busy schedule, a lack of time and space to engage in the intervention for those that followed the training via an app, or unpleasant bodily, mental, or emotional states during mindfulness practice (Supplementary Table S5).

## 4 Discussion

This systematic review of the international scientific literature made it possible to select 49 randomized controlled trials (RCTs) that evaluated the effects of standardized mindfulness programs on burnout. The analysis of RCT’s characteristics shows that they are recent: more than half of the articles have been published in the last three years (53%), overall of good methodological quality. It highlights that 67% of these RCTs showed a statistically significant beneficial effect on at least one of the burnout measurement indicators, where emotional exhaustion being the most impacted aspect.

### 4.1 Effects on burnout

These results provide a strong argument of beneficial effects of mindfulness programs on burnout, given the large number of RCTs included in our systematic review. They are consistent with what is described in the other studies published on the subject (systematic reviews or meta-analyses). These other studies are however less exhaustive and generally include fewer randomized controlled trials. Most of them focus on a single occupational category and evaluate a single type of program. We confirm the results of some other studies from the literature showing the beneficial effects of mindfulness-based programs on burnout in the health sector: medical students (63, 70–72), nurses (66, 73–77), physicians (78–80), or health professionals as a whole (58, 64, 81, 82). We found only one study which, like ours, was carried out on a wide range of professional categories: that of Bartlett et al., carried out in 2019 on 10 studies (61). This meta-analysis measured the effects of mindfulness programs offered to staff by employers in the workplace, but the authors specify that they could not conclude because of the ambivalence of the results.

Regarding the populations studied, the RCTs included in our review involved a total of 7,015 participants, the majority of whom were healthcare professionals (64%). We thus confirm that the care professionals were the most concerned by studies on burnout (59% of RCTs). This can be explained by the close link that has been established, since the creation of the concept of burnout, between the care professions and the symptoms of burnout. Indeed, the first clinical observations of this syndrome were made by psychologist Christina Maslach among healthcare and social service professionals in the United States (83). When she next established the definition of burnout, Christina Maslach suggested that a strong relationship existed between emotional exhaustion, depersonalization, lack of personal fulfillment, and involvement with others (83). In addition, health professionals are most likely one of the professions most affected by burnout, which partly explains the overrepresentation of studies analyzing this socio-professional category (84, 85). It can also be noted that the average percentage of women included in the RCTs of the review is 76%, as already showed by two other studies (59, 81), suggesting that women seem to be more attracted to mindfulness meditative training practices than men., most RCTs having select participants on a voluntary basis.

Our results show that emotional exhaustion was the most positively impacted aspect of burnout when intervened by mindfulness programs. This aspect is a main component of burnout and would result from excessive psychological stress, particularly among professionals devoted to others (83). As mentioned above, the RCTs included in our review have largely studied healthcare professionals who are very prone to burnout, given the demands placed on them and their empathetic abilities (86). Dispositional mindfulness has recently been shown to protect against burnout (87, 88). Also, experiential interventions aimed at promoting mindfulness skills could act in this direction. Mindfulness would act on burnout by promoting the deployment of emotional flexibility in professionals, that is to say their ability to regulate their emotions. Indeed, by practicing mindfulness, professionals develop their attentional and metacognitive capacities as well as emotional flexibility (34, 35, 38, 39, 41, 42). Thus, aware of their emotional state, their limits, and their resources, they preserve themselves better and better regulate their state of stress.

The other two dimensions, depersonalization and personal accomplishment, seem to be only slightly impacted by the mindfulness programs tested. One wonders if programs that include more loving-kindness meditation might better regulate difficult mental states that are typical of depersonalization such as hostility, resentment, withdrawal, or indifference. Indeed, this type of practice promotes the deployment of prosocial qualities of altruism, generosity and love, compassion, and tolerance (34, 42–44). Lasting effects, over several months, could thus promote more empathy and listening among professionals.

### 4.2 Mindfulness programs

Regarding the mindfulness programs evaluated in the studies, our review reveals a great heterogeneity of programs. All the RCTs together assessed the effect of nearly 31 different mindfulness training programs on burnout. We show that the MBSR program accounts for almost a quarter of the interventions tested in the RCTs on burnout (20% including modified MBSR). This result can be explained by the anteriority of MBSR and is consistent with our results showing that the majority (43%) of RCTs conducted on mindfulness and burnout were from the Unites States of America. MBSR was initiated more than 40 years ago in the United States; it was the first standardized program evaluated in the context of interventional research in a therapeutic setting, (28, 31, 89). These figures also agree with those of a recent literature review showing that among 119 countries listed as contributing to the mindfulness literature on Web of Science, USA have the highest research output (46.7%) (29).

Regarding the characteristics of the programs tested, for 35% of the RCTs, the training lasted eight weeks. Unsurprisingly, the mindfulness practices most employed are focused attention and moving practice, which are foundational practices of the MBSR program and of mindfulness practices in general (90). In 73% of the studies, the programs were delivered face-to-face, but a significant number were delivered via a digital medium (18%). This course material is interesting for pursuing a practice of mindfulness at home at any time, unlike face-to-face, which imposes specific schedules on participants. Nevertheless, it has recently been shown that programs delivered face-to-face or digitally would have equivalent effectiveness on mental well-being (32, 33).

Of the 49 RCTs of our systematic review, 21 involved a mindfulness program that lasted at least 16 h. Among them, 18 studies (86%) showed a significant improvement on at least one of the indicators of burnout compared with the control group. Thus, higher the duration of the program, the more it appeared to have a significant effect on burnout. In view of the results, it is interesting to note that there is a minimum number of hours for which the program becomes effective, unlike the recent study of Mikkelsen et al. which shows that post-intervention effectiveness does not seem to depend on the duration of an intervention, except for mental health outcomes (91).

All six RCTs conducted with teachers showed beneficial effects of mindfulness on burnout (one of which tested training lasting less than 16 h). Each of the studies with stressed volunteers, family caregivers, and law enforcement officers was equally effective (two out of three studies had training duration greater than 16 h). In healthcare professionals, 17 of 29 studies (59%) show a significant beneficial effect (of the 12 that showed no effect, 10 tested training lasting less than 16 h). It is difficult to deduce that the profession influences the effect of meditation on burnout because the determining factor of the number of hours of training interferes with the results: in this regard, it should be taken into account that in providing a mindfulness-based intervention to nursing staff (very committed and dedicated), an extended duration of 8 weeks may cause serious practical difficulties, therefore some studies have used shorter versions of the program, finding them equally effective (59).

Regarding the long-term effects, less than half of the studies investigated them and only 10 studies out of 22 showed a significant beneficial effect on burnout indicators. This figure can be explained by the fact that the cessation of the practice of mindfulness for a certain period logically leads to a decrease in acquired skills such as regulation and emotional flexibility, attentional capacities, and therefore burnout. It is likely that meditation is a discipline that requires maintenance and diligent practice to obtain long-term results.

Finally, regarding the adverse effects likely to occur during or after mindfulness programs, a quarter of the studies investigated them (N = 12) but only three studies found small unpleasant effect associated with the practices (busy schedule were mentioned). However, we cannot say that the practice of mindfulness itself is at the root of these feelings; therefore, mindfulness programs are very well tolerated by professionals.

### 4.3 Strengths and limitations of the review

As we detailed above, in view of the other works carried out on the subject, this systematic review is very original on several points. It concerned:

- exclusively RCTs, which are studies considered to have a very high level of scientific evidence, in the context of intervention research.
- a large number of RCTs.
- a large panel of participants belonging to different socio-professional categories.
- several types of standardized mindfulness programs, delivered or not in the workplace (nearly 30 programs have been identified).
- burnout indicators exclusively.

In addition, the selection of articles and the extraction of data were carried out by three investigators.

Some limitations:

- our work is qualitative and not a meta-analysis. The latter could not be carried out given the variety of programs in terms of duration, medium, content, etc.
- all RCT studies included in our review were not blinded. The participants included in the studies were selected on a voluntary basis and were fully aware of the treatment that they were receiving. Additionally, burnout scores (pre- and post-intervention) could not be masked from participants as they used self-administered questionnaires.
- intervention groups with active control were only in a minority.
- most studies were monocentric, where the mindfulness program often delivered by a single instructor.

As we mentioned in the first part of this review, several major obstacles to the search for burnout should also be highlighted: the absence of an established, definitive, and consensual definition which is unanimous (2) and the fact that burnout is not considered an illness (1). This results in difficulties in determining the symptoms and therefore in making a real “diagnosis.”

One of the weaknesses of the studies analyzed is that they claim to have studied the impact of mindfulness programs on burnout when burnout is ultimately not clearly diagnosed. It is important to remember that the identification of burnout is carried out in the clinic by looking for work-related risk factors, individual factors, looking for clinical manifestations via an interrogation and a clinical examination carried out by the general practitioner (or the occupational doctor) (11). All the people selected and included in the studies were considered to be in a state of burnout. The problem with studies is that they use measurement tools as diagnostic tools (13–20). We therefore do not know whether the participants included really suffered from burnout. Is the impact of a program the same on participants with severe burnout and low burnout? Furthermore, the erroneous use of the Maslach Burnout Inventory is problematic: some authors have deleted one or even two scales, others have calculated a total score. These different interpretations did not allow us to analyze the burnout in a homogeneous way (12).

## 5 Conclusion

In the absence of a consensual definition of burnout, it finally seems difficult to be able to study it in intervention research, especially since the tools for evaluating burnout currently proposed do not constitute diagnostic tools. Levels of burnout cannot always be clearly identified, which makes it problematic to study the effectiveness of treatments.

Concerning the evaluation of the effects of mindfulness programs on indicators measuring burnout, the result of our study nevertheless highlights a statistically significant beneficial effect in 67% of randomized controlled trials that have been published over the past ten of years. What seems to emerge from this study is that the duration determines the effect of the programs; indeed, studies exceeding 16 h of training seem to have a beneficial effect on burnout. This information may be of interest in the future design of mindfulness programs to maximize their effectiveness, whatever the type of professionals involved.

Among the 31 mindfulness interventions that have been tested in RCTs, some of them, which include practices of focused attention and open monitoring, are effective on the indicators measuring the emotional component of burnout, suggesting that they could constitute approaches of choice in the prevention of burnout. Nevertheless, it could be appropriate to develop a specific program to impact the three dimensions of burnout. For example, to regulate difficult mental states typical of depersonalization such as hostility, resentment, withdrawal, or indifference, self-compassion and loving-kindness practices could be systematically included in the program. These practices indeed promote the deployment of prosocial qualities of altruism, generosity, compassion, and tolerance.

## Data availability statement

The original contributions presented in the study are included in the article/Supplementary material, further inquiries can be directed to the corresponding author.

## Author contributions

DS: Formal analysis, Writing – original draft. LD: Writing – original draft, Visualization, Formal analysis. NL: Formal analysis, Writing – original draft. AH: Conceptualization, Project administration, Supervision, Validation, Visualization, Writing – original draft, Methodology.
