# Supplementary material for: Effects of standardized mindfulness programs on burnout: a systematic review and original analysis from randomized controlled trials
Source: Front Public Health. 2024 May 22;12:1381373. doi: 10.3389/fpubh.2024.1381373 (PMC11151852; doi:10.3389/fpubh.2024.1381373)
Supplement: Supplementary file 1 [file Data_Sheet_1.docx]

# Table S1. Search strategies for each database.

*EBSCOhost included Academic Search Premier, APA PsycArticles, APA PsycInfo, Business Source Premier, CINAHL Complete, Dentistry & Oral Sciences Source, eBook Collection (EBSCOhost), EconLit, Ergonomics Abstracts, GreenFILE, History of Science, Technology & Medicine, Library, Information Science & Technology Abstracts, MathSciNet via EBSCOhost, Psychology and Behavioral Sciences Collection, Regional Business News, and Teacher Reference Center.

**HAL (short for Hyper Articles en Ligne) is an open access repository of the CCSD (Centre pour la Communication Scientifique Directe), which complies with the Open Archives Initiative-Protocol Metadata Harvesting (OAI-PMH) as well as with the European OpenAIRE project.

| **Database** | **Search strategy** | **Filter** |
| --- | --- | --- |
| EBSCOhost* | ((AB (mindfulness) AND AB (burnout or burn-out or burn out) AND AB (randomized or randomised)) OR ((TI (mindfulness) AND TI (burnout or burn-out or burn out) AND TI (randomized or randomised)) | Peer-reviewed journals  All databases |
| Embase | mindfulness: ab,ti AND (randomised: ab, ti OR randomized: ab, ti) AND (burnout: ab, ti OR ‘burn out’: ab, ti) | Article  Article in press |
| HAL** | mindfulness AND burnout AND randomized | No filter |
| PubMed | (mindfulness[Title/Abstract]) AND ((randomised[Title/Abstract]) OR (randomized[Title/Abstract])) AND ((burnout[Title/Abstract]) OR (burn-out[Title/Abstract]) OR (burn out[Title/Abstract])) | No filter |

# Table S2. Characteristics of the included studies.

Abbreviations: Can. Canada; CG, Control Group; ESRT, Enhanced Stress Resilience Training; F Female; M Male; MG Mindfulness Group; NR Not Reported; PBS Positive Behavior Support;

SD Standard Deviation; SEL Social Emotional Learning; UK United-Kingdom; USA United-States of America; WPE Without Practical Exercise; WSM Web-based Stress Management; WSMg, Web-based Stress Management with weekly group meeting; WSMI Workplace Stress Management Intervention.

| Authors | Publication Year | Country | Participants | Gender % (F/M) | Age  (mean ± SD) | Sample size (MG/CG) | Dropout rate % | Comparison group |
| --- | --- | --- | --- | --- | --- | --- | --- | --- |
| Allexandre et al. WSM | 2016 | USA | Employees | 85/15 | 40 ± 13 | 54/37 | 48 | Waiting list |
| Allexandre et al. WSMg | 2016 | USA | Employees | 82/18 | 40 ± 12 | 70/37 | 50 | Waiting list |
| Ameli et al. | 2020 | USA | Health care professionals | 83/17 | 32 | 45/37 | 15 | Waiting list |
| Amutio et al. | 2015 | Spain | Health care professionals | 57/43 | 47 ± 9 | 21/21 | 0 | Waiting list |
| Arredondo et al. | 2017 | Spain | Employees | 78/22 | 37 ± 6 | 21/19 | 18 | Waiting list |
| Asuero et al. | 2014 | Spain | Health care professionals | 92/8 | 47 ± 8 | 43/25 | 0 | Waiting list |
| Braun et al. | 2020 | USA | Health care students | 92/8 | 26 ± 5 | 22/26 | 33 | Waiting list |
| Cascales-Pérez et al. | 2021 | Spain | Health care professionals | 81/19 | 51 ± 10 | 30/28 | 0 | Single theoretical session |
| Christopher et al. | 2018 | USA | Law enforcement officers | 11/89 | 44 ± 6 | 31/30 | 20 | No intervention |
| Chu et al. | 2022 | USA | Health care students | 73/27 | NR | 44/43 | 36 | Waiting list |
| De Carvalho et al. | 2021 | Portugal | Teachers | 96/4 | 44 ± 7 | 123/105 | 10 | Waiting list |
| De Vibe et al. | 2013 | Norway | Health care students | 76/24 | 24 ± 5 | 144/144 | 4 | No intervention |
| Duchemin et al. | 2015 | USA | Health care professionals | 88/12 | 44 | 16/16 | 0 | Waiting list |
| Eriksson et al. | 2018 | Sweden | Health care professionals | 97/3 | 36 ± 8 | 52/49 | 20 | Waiting list |
| Fendel et al. | 2021 | Germany | Health care professionals | 65/35 | 31 ± 3 | 77/73 | 19 | Coursebook WPE |
| Fischer et al. | 2022 | Germany | Stressed volunteers | 89/11 | 47 ± 12 | 34/33 | 19 | Iyengar Yoga |
| Flook et al. | 2013 | USA | Teachers | 89/11 | 43 ± 10 | 10/8 | 0 | Waiting list |
| Fraiman et al. | 2022 | USA | Health care professionals | 75/25 | NR | 194/146 | 43 | Social lunches |
| Haghighinejad et al. | 2022 | Iran | Health care professionals | 52/48 | 37 ± 6 | 25/25 | 0 | No intervention |
| Hilcove et al. | 2021 | USA | Health care professionals | 95/5 | 42 | 41/39 | 3 | No intervention |
| Ireland et al. | 2017 | Australia | Health care professionals | 64/36 | 27 ± 5 | 23/21 | 0 | Extra break sessions |
| Jennings | 2013 | USA | Teachers | 89/11 | 36 | NR/NR | 6 | Waiting list |
| Kukihara et al. | 2022 | Japan | Health care professionals | 83/17 | 47 ± 12 | 17/12 | 7 | No intervention |
| Kuyken et al. | 2022 | UK | Teachers | 75/25 | 40 ± 9 | 362/317 | NR | SEL |
| Lebares et al. (ESRT1) | 2021 | USA | Health care professionals | 50/50 | 28 ± 2 | 23/21 | 9 | Active listening and reading about medicine |
| Lebares et al. (ESRT2) | 2021 | USA | Health care professionals | 47/53 | 29 ± 2 | 23/22 | 0 |  |
| Lebares et al. | 2019 | USA | Health care professionals | 38/62 | 28 ± 2 | 12/9 | 0 |  |
| Mackenzie et al. | 2006 | NR | Health care professionals | 97/3 | 47 ± 7 | 16/14 | 0 | Waiting list |
| Martínez-Borrás et al. | 2022 | Spain | Employees | 42/58 | 42 ± 10 | 20/20 | 40 | WSMI |
| Mistretta et al. | 2018 | USA | Health care professionals | 92/8 | 47 ± 11 | 22/15 | 30 | Waiting list |
| Moody, K et al. | 2013 | USA & Israel | Health care professionals | 80/20 | NR | 23/24 | 2 | No intervention |
| O'Driscoll et al. An online | 2019 | Ireland | Health care students | 77/23 | NR | 68/71 | 63 | Waiting list |
| O'Driscoll et al. Impact | 2019 | Ireland | Health care students | 67/33 | NR | 81/83 | 40 | Waiting list |
| Pérez et al. | 2022 | Spain | Health care professionals | 90/10 | 37 ± 9 | 39/35 | 0 | Waiting list |
| Prudenzi et al. | 2022 | UK | Health care professionals | 93/7 | 43 ± 10 | 65/54 | 47 | Waiting list |
| Purdie et al. | 2022 | USA | Health care professionals | 79/21 | NR | 27/39 | 5 | Waiting list |
| Roeser et al. | 2013 | USA & Can. | Teachers | 88/12 | 47 ± 9 | 60/59 | 5 | Waiting list |
| Sairanen et al. | 2020 | Sweden | Family caregivers | 81/19 | 43 ± 7 | 37/37 | 38 | Waiting list |
| Schroeder et al. | 2018 | USA | Health care professionals | 73/27 | 43 ± 8 | 16/17 | 21 | Waiting list |
| Seidel et al. | 2021 | USA | Health care professionals | 86/14 | NR | NR/NR | 19 | Waiting list |
| Singh et al. Using | 2020 | NR | Health care professionals | 56/44 | 40 | 72/72 | 8 | Training as usual |
| Singh et al. Comparative | 2020 | NR | Health care professionals | 71/29 | 43 ± 9 | 60/63 | 6 | PBS |
| Smith et al. | 2021 | USA | Health care students | 79/21 | NR | 8/8 | 0 | Waiting list |
| Strub & Tarquinio | 2013 | Luxembourg | Employees | 40/60 | NR | 10/10 | 0 | No intervention |
| Taylor et al. | 2022 | UK | Health care professionals | 83/17 | 41 ± 11 | 1095/1087 | 49 | Moodzone |
| Verweij et al. | 2018 | Netherlands | Health care professionals | 88/12 | 31 ± 5 | 80/68 | 7 | Waiting list |
| Watanabe et al. | 2019 | Japan | Health care professionals | 100/0 | 30 ± 8 | 40/40 | 5 | Psychoeducation leaflet |
| Xu et al. | 2022 | Australia | Health care professionals | 78/22 | NR | 74/74 | 26 | Waiting list |
| Zheng et al. | 2022 | China | Teachers | 89/11 | 33 ± 8 | 83/73 | 38 | Waiting list |

# Table S3. Quality assessment of the 49 randomized controlled trials included in the review (using the part 2 of the Mixed Methods Appraisal Tool MMAT)

| Authors of the studies | Methodological quality criteria | | | | | |
| --- | --- | --- | --- | --- | --- | --- |
|  | **1** | **2** | **3** | **4** | **5** | |
|  | **Randomi-zation** | **Baseline** | **Complete data** | **Blinding** | | **Adherence** |
| Allexandre et al. (2016) (WSM) | Y | C | N | N | N | |
| Allexandre et al. (2016) (WSMg) | Y | C | N | N | N | |
| Ameli et al. (2020) | Y | Y | Y | N | Y | |
| Amutio et al. (2015) | Y | Y | Y | N | Y | |
| Arredondo et al. (2017) | Y | Y | Y | N | Y | |
| Asuero et al. (2014) | Y | Y | Y | N | Y | |
| Braun et al. (2020) | Y | Y | N | N | N | |
| Cascales-Pérez et al. (2021) | Y | C | Y | N | Y | |
| Christopher et al. (2018) | Y | N | N | C | Y | |
| Chu et al. (2022) | Y | Y | N | N | Y | |
| De Carvalho et al. (2021) | N | N | Y | N | Y | |
| De Vibe et al. (2013) | Y | N | Y | N | N | |
| Duchemin et al. (2015) | Y | Y | Y | N | Y | |
| Eriksson et al. (2018) | N | C | N | N | Y | |
| Fendel et al. (2021) | Y | N | Y | N | Y | |
| Fischer et al. (2022) | Y | Y | Y | N | Y | |
| Flook et al. (2013) | N | Y | Y | N | Y | |
| Fraiman et al. (2022) | Y | N | N | N | Y | |
| Haghighinejad et al. (2022) | Y | Y | Y | N | Y | |
| Hilcove et al. (2021) | Y | Y | Y | N | Y | |
| Ireland et al. (2017) | N | C | Y | C | Y | |
| Jennings (2013) | N | Y | Y | N | Y | |
| Kukihara et al. (2022) | N | Y | Y | C | Y | |
| Kuyken et al. (2022) | Y | N | N | N | Y | |
| Lebares et al. (2021) (ESRT1) | Y | N | Y | Y | N | |
| Lebares et al. (2021) (ESRT2) | Y | N | Y | Y | N | |
| Lebares et al. (2019) | Y | N | Y | Y | N | |
| Mackenzie et al. (2006) | N | N | Y | N | Y | |
| Martínez-Borrás et al. (2022) | Y | N | N | C | Y | |
| Mistretta et al. (2018) | Y | Y | N | C | Y | |
| Moody, K et al. (2013) | Y | N | Y | N | Y | |
| O'Driscoll et al. (2019) An online | N | Y | N | N | Y | |
| O'Driscoll et al. (2019) Impact | N | Y | N | N | Y | |
| Pérez et al. (2022) | N | Y | Y | N | Y | |
| Prudenzi et al. (2022) | Y | Y | N | N | N | |
| Purdie et al. (2022) | Y | Y | Y | N | Y | |
| Roeser et al. (2013) | N | N | Y | N | Y | |
| Sairanen et al. (2020) | Y | C | N | N | Y | |
| Schroeder et al. (2018) | N | Y | N | N | Y | |
| Seidel et al. (2021) | N | C | Y | N | C | |
| Singh et al. (2020) Using | N | N | Y | N | Y | |
| Singh et al. (2020) Comparative | Y | C | Y | C | Y | |
| Smith et al. (2021) | Y | Y | Y | N | N | |
| Strub & Tarquinio (2013) | N | N | Y | N | Y | |
| Taylor et al. (2022) | Y | Y | N | Y | Y | |
| Verweij et al. (2018) | Y | N | Y | N | Y | |
| Watanabe et al. (2019) | Y | C | Y | N | Y | |
| Xu et al. (2022) | Y | Y | N | N | Y | |
| Zheng et al. (2022) | Y | N | N | N | N | |

**QUALITY CRITERIA OF RCTs**

# Figure S1 Methodological quality assessment of randomized controlled trials (RCTs, n=49) with 5 criteria (using the part 2 of the MMAT).

**A**. The authors described how the randomization schedule was generated in 69% of studies. **B.** In 47% of studies, authors specified that the groups were comparable at baseline; 35% found initial significant differences between the groups (sociodemographic characteristics or outcomes). **C**. In 63% of studies collection of the data was completed, the dropout rate was less than 20%. **D.** In 80% of studies, participants (outcome assessors) were not blinded to the condition assignment. **E.** In 78% of studies, participants received their assigned intervention, while those in 20% did not, for different reasons (schedule conflict, switches to another active intervention, …).

# Table S4. Mindfulness training characteristics.

Abbreviations: MBSR Mindfulness-Based Stress Reduction; MBCT Mindfulness-Based Cognitive Therapy; SBMT School-Based Mindfulness Training; FA Foccused Attention; OM Open Monitoring; NR Not Reported.

| Authors | Mindfulness training | Mindfulness practices | | | | Duration/Intensity of mindfulness training | Number of instructors | Training materials |
| --- | --- | --- | --- | --- | --- | --- | --- | --- |
|  |  | **FA** | **Self-care** | **OM** | **Moving** |  |  |  |
| Allexandre et al. (2016) (WSM) | Web stress management | Yes | Yes | NR | No | Access to the application 35 min 5 times a week for 8 weeks | NR | Digital media |
| Allexandre et al. (2016) (WSMg) | Web stress management, with Weekly Group Meeting (and Expert Clinical Support) | Yes | Yes | NR | No | Access to the application 35 min 5 times a week for 8 weeks + 8 weekly sessions of 1h, with or without the intervention of an expert | 2 | On site and digital media |
| Ameli et al. (2020) | Mindfulness-Based Self-Care (MBSC) | Yes | Yes | NR | Yes | 5 weekly sessions of 1,5h | 1 | On site |
| Amutio et al. (2015) | MBSR | Yes | No | Yes | Yes | 8 weekly sessions of 2,5h + 8-hour retreat | 1 | On site |
| Arredondo et al. (2017) | Unnamed program | Yes | Yes | Yes | NR | 8 weekly sessions of 1,5 h + 3-hour retreat | 1 | On site |
| Asuero et al. (2014) | MBSR | Yes | No | Yes | Yes | 8 weekly sessions of 2,5h + 8-hour intensive session | 1 | On site |
| Braun et al. (2020) | Mindfulness for Interdisciplinary Healthcare Professionals (MIHP) | Yes | Yes | No | Yes | 8 weekly sessions of 2h | 2 | On site |
| Cascales-Pérez et al. (2021) | Modified MBSR | Yes | Yes | No | Yes | 8 weekly sessions of 2,5h | 1 | On site |
| Christopher et al. (2018) | Mindfulness-Based Resilience Training (MBRT) | Yes | No | No | Yes | 8 weekly sessions of 2h + extended 6-hour class in the seventh week | Several | On site |
| Chu et al. (2022) | Headspace | Yes | NR | NR | NR | 10 minutes a day for 6 weeks | 1 | Digital media |
| De Carvalho et al. (2021) | Atentamente | Yes | Yes | Yes | No | 10 weekly sessions of 2,5h + 5-hour booster session 3 months later | 1 | On site |
| De Vibe et al. (2013) | Modified MBSR | Yes | NR | Yes | Yes | 6 weekly sessions of 1,5h + 6-hour session | 6 | On site |
| Duchemin et al. (2015) | Unnamed program | Yes | No | No | Yes | 8 weekly sessions of 1h + extended 2-hour session in the fifth week | 1 | On site |
| Eriksson et al. (2018) | Mindfulness and compassion with self and others | Yes | Yes | No | No | 15 min 6 times a week for 6 weeks | NR | Digital media |
| Fendel et al. (2021) | A tailored Mindfulness-Based Program for residents | Yes | Yes | Yes | Yes | 8 weekly sessions of 2,25h + 6-hour retreat + 4-month maintenance phase (3 monthly booster sessions of 2,5h) | 3 | On site |
| Fischer et al. (2022) | Unnamed program | Yes | Yes | No | No | 12 weekly sessions of 1,5h | Several | Online |
| Flook et al. (2013) | Modified MBSR for teachers | Yes | Yes | Yes | Yes | 8 weekly sessions of 2,5h + 6-hour session | 2 | On site |
| Fraiman et al. (2022) | Mindfulness Intervention for New Interns (MINdI) | Yes | No | No | Yes | 7 sessions of 1h over 6 months | 1 | On site |
| Haghighinejad et al. (2022) | modified MBSR | Yes | No | No | No | 4 weekly sessions of 1h | 2 | On site |
| Hilcove et al. (2021) | Mindfulness-Based Yoga Intervention | Yes | No | No | Yes | 6 weekly sessions | NR | On site |
| Ireland et al. (2017) | Unnamed program | Yes | NR | No | No | 10 weekly sessions of 1h | NR | On site |
| Jennings (2013) | CARE: Cultivating Awareness and Resilience in Education | Yes | Yes | Yes | Yes | 2-day weekend session (12 hours) followed by a 6-hour session 2 weeks later and a 6-hour session 4 weeks after the initial sessions + 6-hour booster session 1 month later | Several | On site |
| Kukihara et al. (2022) | Unnamed program | Yes | NR | NR | No | 6 weekly sessions of 1h | 1 | On site |
| Kuyken et al. (2022) | School-based mindfulness training (SBMT) | Yes | NR | Yes | Yes | 1 year (8 weekly MBCT-L sessions of 2h + 4-day training workshop to learn how to deliver SBMT to students + 2 SBMT programs of 10 lessons of 30-50 min each, over one school term) | NR | On site |
| Lebares et al. (2021) (ESRT1) | Enhanced Stress Resilience Training 1 | Yes | No | Yes | Yes | 8 weekly sessions of 2h + 3-hour retreat | 1 | On site |
| Lebares et al. (2021) (ESRT2) | Enhanced Stress Resilience Training 2 | Yes | No | Yes | Yes | 6 weekly sessions of 1,5h + 3-hour retreat | 1 | On site |
| Lebares et al. (2019) | Modified MBSR | Yes | No | Yes | Yes | 8 weekly sessions of 2h + 3-hour retreat | 1 | On site |
| Mackenzie et al. (2006) | Modified MBSR | Yes | No | No | Yes | 4 weekly sessions of 30 minutes | NR | On site |
| Martínez-Borrás et al. (2022) | Mindfulness and Self-Compassion-Based Intervention (MSCBI) | Yes | Yes | Yes | Yes | 6 weekly sessions for a total of 12 hours | 1 | On site |
| Mistretta et al. (2018) | Mindfulness-Based Resilience Training (MBRT) | Yes | Yes | No | Yes | 6 weekly sessions of 2h | 1 | On site |
| Moody, K et al. (2013) | Mindfulness-Based Course (MBC) | Yes | Yes | Yes | Yes | First session of 6h + 6 weekly sessions of 1h + 1 final session of 3h | 2 | On site |
| O'Driscoll et al. (2019) An online | Online Mindfulness-Based Intervention | Yes | No | Yes | Yes | 4 weekly online sessions of 1h | 1 | Digital media |
| O'Driscoll et al. (2019) Impact | Modified MBSR | Yes | No | Yes | Yes | 4 weekly sessions of 2h | 1 | On site |
| Pérez et al. (2022) | Unnamed program | Yes | NR | NR | NR | 6 weekly recorded sessions of 1h | 2 | Digital media |
| Prudenzi et al. (2022) | Acceptation and Commitment Therapy (ACT) | Yes | No | Yes | No | 4 weekly sessions of 2h | 3 | On site |
| Purdie et al. (2022) | Hybrid Mindful Awareness Practices | Yes | Yes | No | Yes | 1-hour teacher-delivered session + 6 weeks of access to the digital format | 1 | On site and digital media |
| Roeser et al. (2013) | Unnamed program | Yes | Yes | Yes | Yes | 11 sessions over 8 weeks (6-hour session + 9 sessions of 2,5h + 6-hour retreat) | 1 | On site |
| Sairanen et al. (2020) | Web-based ACT | Yes | Yes | Yes | No | 10 weeks | 17 | Digital media |
| Schroeder et al. (2018) | Mindful Medicine Curriculum | Yes | Yes | No | Yes | 13-hour weekend training + 2-hour follow-up sessions scheduled at 2 and 4 weeks after the weekend | Several | On site |
| Seidel et al. (2021) | Building a Mindful CommUnity | Yes | Yes | No | Yes | 4 weekly sessions of 1h | 1 | On site |
| Singh et al. (2020) Using | 3-day Mindfulness-Based Positive Behavior Support (MBPBS) | Yes | Yes | Yes | Yes | 3 consecutive days of 8 h + 32-week formal implementation | 1 | On site |
| Singh et al. (2020) Comparative | 7-day Mindfulness-Based Positive Behavior Support (MBPBS) | Yes | Yes | Yes | Yes | 8-hour day (week 1) + 5 days of 8h (week 5) + 8-hour day (week 10) + 30 weeks (informal meditation practices) | 1 | On site |
| Smith et al. (2021) | 10% Happier | Yes | No | No | No | In theory, 12 minutes a day for 8 weeks Mean = 182,8 min over 8 weeks | NR | Digital media |
| Strub & Tarquinio (2013) | Modified MBCT | Yes | No | No | Yes | 4 sessions of 4 hours (2 sessions per month) | 1 | On site |
| Taylor et al. (2022) | Headspace | Yes | NR | NR | NR | In theory, 10 min per day for 30 days then 90 days | 1 | Digital media |
| Verweij et al. (2018) | MBSR | Yes | No | Yes | Yes | 8 weekly sessions of 2,5h + 6-hour silent day | 11 | On site |
| Watanabe et al. (2019) | Brief Mindfulness-Based Stress Management Program | Yes | No | No | No | 4 weekly sessions of 30 minutes | 25 | On site |
| Xu et al. (2022) | Headspace | Yes | NR | NR | NR | In theory, 10 min per day for 4 weeks. Mean: 2 days/week and 6 min/session | 1 | Digital media |
| Zheng et al. (2022) | Mindfulness-Based Positive Psychology (MBPP) | Yes | Yes | No | No | 6 weekly online sessions of 2,5h | 1 | Online |

# Table S5. Mindfulness training effect on burnout indicators.

Abbreviations: DP Depersonalization; EE Emotional Exhaustion; ESRT Enhanced Stress Resilience Training; MBI Maslach Burnout Inventory; NA Not Applicable; Not rated means that effect of mindfulness training on burnout indicator did not evaluate after intervention but at follow-up only; PA Personal Accomplishment; WSM Web-based Stress Management; WSMg Web-based Stress Management group.

Statistics specified by the authors: *, p ≤ 0.05; **, p ≤ 0,01; ***, p ≤ 0,001; -, No effect.

| Authors of the studies | Burnout scale used | Burnout indicators assessed | | | Burnout total score | Mindfulness program effect | Mindfulness program effect at follow-up (on burnout indicators) |
| --- | --- | --- | --- | --- | --- | --- | --- |
|  |  | **EE** | **DP** | **PA** |  |  |  |
| Allexandre et al. (2016) (WSM) | adapted MBI | - | NA | - | NA | No effect | No effect at 8 weeks |
| Allexandre et al. (2016) (WSMg) | adapted MBI | ** | NA | * | NA | Significant beneficial effect | Significant beneficial effect at 8 weeks (EE) |
| Ameli et al. (2020) | adapted MBI | - | - | NA | NA | No effect  No side effect | NA |
| Amutio et al. (2015) | MBI | * | - | - | * | Significant beneficial effect | NA |
| Arredondo et al. (2017) | adapted MBI | ** | * | * | NA | Significant beneficial effect | Significant beneficial effect at 12 weeks (EE, DP, AP) |
| Asuero et al. (2014) | MBI | ** | * | - | * | Significant beneficial effect | NA |
| Braun et al. (2020) | adapted MBI | * | - | - | NA | Significant beneficial effect | NA |
| Cascales-Pérez et al. (2021) | MBI, Professional Quality of Life scale | *** | ** | - | *** | Significant beneficial effect | NA |
| Christopher et al. (2018) | The Oldenburg Burnout Inventory | NA | NA | NA | ** | Significant beneficial effect | No effect at 13 weeks |
| Chu et al. (2022) | MBI | ** | ** | * | NA | Significant beneficial effect | Significant beneficial effect at 4 weeks (AP) |
| De Carvalho et al. (2021) | adapted MBI | *** | *** | NA | NA | Significant beneficial effect | NA |
| De Vibe et al. (2013) | adapted MBI | NA | NA | NA | - | No effect  No side effect but unpleasant experience | NA |
| Duchemin et al. (2015) | MBI, Professional Quality of Life scale | - | - | - | NA | No effect | NA |
| Eriksson et al. (2018) | The Shirom-Melamed Burnout Questionnaire | NA | NA | NA | ** | Significant beneficial effect | NA |
| Fendel et al. (2021) | The Copenhagen Burnout Inventory | NA | NA | NA | * | Significant beneficial effect | No effect at 26 weeks |
| Fischer et al. (2022) | MBI | - | * | - | NA | Significant beneficial effect  No side effect | Significant beneficial effect at 12 weeks (DP) |
| Flook et al. (2013) | MBI | * | - | * | - | Significant beneficial effect | NA |
| Fraiman et al. (2022) | MBI | - | - | - | NA | No effect | No effect at 39 weeks |
| Haghighinejad et al. (2022) | The Copenhagen Burnout Inventory | NA | NA | NA | * | Significant beneficial effect | Significant beneficial effect at 13 weeks (personal burnout) |
| Hilcove et al. (2021) | MBI | NA | NA | NA | ** | Significant beneficial effect  No side effect | NA |
| Ireland et al. (2017) | The Copenhagen Burnout Inventory | NA | NA | NA | * | Significant beneficial effect | NA |
| Jennings (2013) | MBI | - | - | * | NA | Significant beneficial effect | NA |
| Kukihara et al. (2022) | adapted MBI | ** | - | * | NA | Significant beneficial effect | NA |
| Kuyken et al. (2022) | MBI | ** | - | * | NA | Significant beneficial effect  No side effect | No effect at 52 weeks |
| Lebares et al. (2021) (ESRT1) | adapted MBI | - | - | NA | NA | No effect | No effect at 42 weeks |
| Lebares et al. (2021) (ESRT2) | adapted MBI | * | - | NA | NA | Significant beneficial effect | Significant beneficial effect at 26 weeks (EE, DP) |
| Lebares et al. (2019) | adapted MBI | NA | NA | NA | Not rated | No effect | No effect at 6.5 weeks |
| Mackenzie et al. (2006) | MBI | * | * | - | NA | Significant beneficial effect | NA |
| Martínez-Borrás et al. (2022) | adapted MBI | *** | - | - | NA | Significant beneficial effect | NA |
| Mistretta et al. (2018) | MBI | - | - | - | NA | No effect | No effect at 13 weeks |
| Moody, K et al. (2013) | MBI | - | - | - | NA | No effect  Side effect: busy schedule | NA |
| O'Driscoll et al. (2019) An online | adapted MBI | - | - | ** | NA | Significant beneficial effect  No side effect | NA |
| O'Driscoll et al. (2019) Impact | adapted MBI | - | - | - | NA | No effect  No side effect | NA |
| Pérez et al. (2022) | Professional Quality of Life scale | NA | NA | NA | *** | Significant beneficial effect | Significant beneficial effect at 13 weeks (total score) |
| Prudenzi et al. (2022) | The Shirom-Melamed Burnout Measure | NA | NA | NA | * | Significant beneficial effect | Significant beneficial effect at 10 weeks (total score) |
| Purdie et al. (2022) | adapted MBI | - | - | - | NA | No effect  No side effect | NA |
| Roeser et al. (2013) | MBI | NA | NA | NA | ** | Significant beneficial effect | Significant beneficial effect at 13 weeks (total score) |
| Sairanen et al. (2020) | The Shirom-Melamed Burnout Questionnaire | NA | NA | NA | ** | Significant beneficial effect | No effect at 17 weeks |
| Schroeder et al. (2018) | MBI | * | * | - | NA | Significant beneficial effect | Significant beneficial effect at 13 weeks (EE, DP) |
| Seidel et al. (2021) | MBI | - | - | - | NA | No effect | No effect at 26 weeks |
| Singh et al. (2020) Using | Professional Quality of Life scale | NA | NA | NA | *** | Significant beneficial effect | NA |
| Singh et al. (2020) Comparative | Professional Quality of Life scale | NA | NA | NA | *** | Significant beneficial effect | NA |
| Smith et al. (2021) | School Burnout Inventory | NA | NA | NA | - | No effect  No side effect | NA |
| Strub & Tarquinio (2013) | MBI | * | - | - | NA | Significant beneficial effect | NA |
| Taylor et al. (2022) | MBI | - | - | - | NA | No effect  Side effect : busy schedule | NA |
| Verweij et al. (2018) | adapted MBI | - | - | * | NA | Significant beneficial effect | NA |
| Watanabe et al. (2019) | MBI | Not rated | Not rated | Not rated | NA | No effect  Any serious adverse event | No effect at 9 weeks |
| Xu et al. (2022) | MBI | - | - | - | NA | No effect | No effect at 13 weeks |
| Zheng et al. (2022) | adapted MBI | * | *** | - | *** | Significant beneficial effect | NA |

# REFERENCES OF THE ARTICLES INCLUDED IN THE SYSTEMATIC REVIEW

Allexandre D, Bernstein AM, Walker E, Hunter J, Roizen MF, Morledge TJ. A Web-Based Mindfulness Stress Management Program in a Corporate Call Center: A Randomized Clinical Trial to Evaluate the Added Benefit of Onsite Group Support. J Occup Environ Med. 2016;58: 254–264. doi:[10.1097/JOM.0000000000000680](https://doi.org/10.1097/JOM.0000000000000680)

Ameli R, Sinaii N, West CP, Luna MJ, Panahi S, Zoosman M, et al. Effect of a Brief Mindfulness-Based Program on Stress in Health Care Professionals at a US Biomedical Research Hospital: A Randomized Clinical Trial. JAMA Netw Open. 2020;3: e2013424. doi:[10.1001/jamanetworkopen.2020.13424](https://doi.org/10.1001/jamanetworkopen.2020.13424)

Amutio A, Martínez-Taboada C, Delgado LC, Hermosilla D, Mozaz MJ. Acceptability and Effectiveness of a Long-Term Educational Intervention to Reduce Physicians’ Stress-Related Conditions. J Contin Educ Health Prof. 2015;35: 255–260. doi:[10.1097/CEH.0000000000000002](https://doi.org/10.1097/CEH.0000000000000002)

Arredondo M, Sabaté M, Valveny N, Langa M, Dosantos R, Moreno J, et al. A mindfulness training program based on brief practices (M-PBI) to reduce stress in the workplace: a randomised controlled pilot study. Int J Occup Environ Health. 2017;23: 40–51. doi:[10.1080/10773525.2017.1386607](https://doi.org/10.1080/10773525.2017.1386607)

Asuero AM, Queraltó JM, Pujol-Ribera E, Berenguera A, Rodriguez-Blanco T, Epstein RM. Effectiveness of a mindfulness education program in primary health care professionals: a pragmatic controlled trial. J Contin Educ Health Prof. 2014;34: 4–12. doi:[10.1002/chp.21211](https://doi.org/10.1002/chp.21211)

Braun SE, Dow A, Loughan A, Mladen S, Crawford M, Rybarczyk B, et al. Mindfulness training for healthcare professional students: A waitlist controlled pilot study on psychological and work-relevant outcomes. Complementary Therapies in Medicine. 2020;51: 102405. doi:[10.1016/j.ctim.2020.102405](https://doi.org/10.1016/j.ctim.2020.102405)

Cascales-Pérez ML, Ferrer-Cascales R, Fernández-Alcántara M, Cabañero-Martínez MJ. Effects of a mindfulness-based programme on the health- and work-related quality of life of healthcare professionals. Scand J Caring Sci. 2021;35: 881–891. doi:[10.1111/scs.12905](https://doi.org/10.1111/scs.12905)

Christopher MS, Hunsinger M, Goerling LtRJ, Bowen S, Rogers BS, Gross CR, et al. Mindfulness-based resilience training to reduce health risk, stress reactivity, and aggression among law enforcement officers: A feasibility and preliminary efficacy trial. Psychiatry Research. 2018;264: 104–115. doi:[10.1016/j.psychres.2018.03.059](https://doi.org/10.1016/j.psychres.2018.03.059)

Chu A, Rose TM, Gundrum DA, McMorris TE, Klausner EA, Lang LA, et al. Evaluating the effects of a mindfulness mobile application on student pharmacists’ stress, burnout, and mindfulness. Am J Health-Syst Pharm. 2022;79: 656–664. doi:[10.1093/ajhp/zxab467](https://doi.org/10.1093/ajhp/zxab467)

De Carvalho JS, Oliveira S, Roberto MS, Gonçalves C, Bárbara JM, De Castro AF, et al. Effects of a Mindfulness-Based Intervention for Teachers: a Study on Teacher and Student Outcomes. Mindfulness. 2021;12: 1719–1732. doi:[10.1007/s12671-021-01635-3](https://doi.org/10.1007/s12671-021-01635-3)

De Vibe M, Solhaug I, Tyssen R, Friborg O, Rosenvinge JH, Sørlie T, et al. Mindfulness training for stress management: a randomised controlled study of medical and psychology students. BMC Med Educ. 2013;13: 107. doi:[10.1186/1472-6920-13-107](https://doi.org/10.1186/1472-6920-13-107)

Duchemin A-M, Steinberg BA, Marks DR, Vanover K, Klatt M. A small randomized pilot study of a workplace mindfulness-based intervention for surgical intensive care unit personnel: Effects on salivary α-amylase levels. J Occup Environ Med. 2015;57: 393–399. doi:[10.1097/JOM.0000000000000371](https://doi.org/10.1097/JOM.0000000000000371)

Eriksson T, Germundsjö L, Åström E, Rönnlund M. Mindful self-compassion training reduces stress and burnout symptoms among practicing psychologists: A randomized controlled trial of a brief web-based intervention. Frontiers in Psychology. 2018;9. doi:[10.3389/fpsyg.2018.02340](https://doi.org/10.3389/fpsyg.2018.02340)

Fendel JC, Aeschbach VM, Schmidt S, Göritz AS. The impact of a tailored mindfulness-based program for resident physicians on distress and the quality of care: A randomised controlled trial. J Intern Med (GBR). 2021;290: 1233–1248. doi:[10.1111/joim.13374](https://doi.org/10.1111/joim.13374)

Fischer JM, Kandil F-I, Kessler CS, Nayeri L, Zager LS, Rocabado Hennhöfer T, et al. Stress Reduction by Yoga versus Mindfulness Training in Adults Suffering from Distress: A Three-Armed Randomized Controlled Trial including Qualitative Interviews (RELAX Study). J Clin Med. 2022;11. doi:[10.3390/jcm11195680](https://doi.org/10.3390/jcm11195680)

Flook L, Goldberg SB, Pinger L, Bonus K, Davidson RJ. Mindfulness for teachers: A pilot study to assess effects on stress, burnout, and teaching efficacy. Mind, Brain, and Education. 2013;7: 182–195. doi:[10.1111/mbe.12026](https://doi.org/10.1111/mbe.12026)

Fraiman YS, Cheston CC, Cabral HJ, Allen C, Asnes AG, Barrett JT, et al. Effect of a Novel Mindfulness Curriculum on Burnout during Pediatric Internship: A Cluster Randomized Clinical Trial. JAMA Pediatr. 2022;176: 365–372. doi:[10.1001/jamapediatrics.2021.5740](https://doi.org/10.1001/jamapediatrics.2021.5740)

Haghighinejad H, Ghazipoor H, Jafari P, Taghipour K, Rezaie M, Liaghat L, et al. Investigating the impact of modified mindfulness-based stress reduction (MBSR) program on occupational burnout and other mental health status among nonmedical staff in a hospital: a randomized controlled trial. Int Arch Occup Environ Health. 2022;95: 2005–2016. doi:[10.1007/s00420-022-01902-3](https://doi.org/10.1007/s00420-022-01902-3)

Hilcove K, Marceau C, Thekdi P, Larkey L, Brewer MA, Jones K. Holistic Nursing in Practice: Mindfulness-Based Yoga as an Intervention to Manage Stress and Burnout. J Holist Nurs. 2021;39: 29–42. doi:[10.1177/0898010120921587](https://doi.org/10.1177/0898010120921587)

Ireland MJ, Clough B, Gill K, Langan F, O’Connor A, Spencer L. A randomized controlled trial of mindfulness to reduce stress and burnout among intern medical practitioners. Med Teach. 2017;39: 409–414. doi:[10.1080/0142159X.2017.1294749](https://doi.org/10.1080/0142159X.2017.1294749)

Jennings PA, Frank JL, Snowberg KE, Coccia MA, Greenberg MT. Improving classroom learning environments by Cultivating Awareness and Resilience in Education (CARE): Results of a randomized controlled trial. School Psychology Quarterly. 2013;28: 374–390. doi:[10.1037/spq0000035](https://doi.org/10.1037/spq0000035)

Kukihara H, Ando M, Yamawaki N. The effects of yoga and mindful meditation on elderly care worker’s burnout: a CONSORT-compliant randomized controlled trial. J Rural Med. 2022;17: 14–20. doi:[10.2185/jrm.2021-021](https://doi.org/10.2185/jrm.2021-021)

Kuyken W, Ball S, Crane C, Ganguli P, Jones B, Montero-Marin J, et al. Effectiveness of universal school-based mindfulness training compared with normal school provision on teacher mental health and school climate: results of the MYRIAD cluster randomised controlled trial. Evid Based Mental Health. 2022;25: 125–134. doi:[10.1136/ebmental-2022-300424](https://doi.org/10.1136/ebmental-2022-300424)

Lebares CC, Coaston TN, Delucchi KL, Guvva EV, Shen WT, Staffaroni AM, et al. Enhanced Stress Resilience Training in Surgeons: Iterative Adaptation and Biopsychosocial Effects in 2 Small Randomized Trials. Ann Surg. 2021;273: 424–432. doi:[10.1097/SLA.0000000000004145](https://doi.org/10.1097/SLA.0000000000004145)

Lebares CC, Guvva EV, Olaru M, Sugrue LP, Staffaroni AM, Delucchi KL, et al. Efficacy of Mindfulness-Based Cognitive Training in Surgery: Additional Analysis of the Mindful Surgeon Pilot Randomized Clinical Trial. JAMA Netw Open. 2019;2: e194108. doi:[10.1001/jamanetworkopen.2019.4108](https://doi.org/10.1001/jamanetworkopen.2019.4108)

Mackenzie CS, Poulin PA, Seidman-Carlson R. A brief mindfulness-based stress reduction intervention for nurses and nurse aides. Appl Nurs Res. 2006;19: 105–109. doi:[10.1016/j.apnr.2005.08.002](https://doi.org/10.1016/j.apnr.2005.08.002)

Martínez-Borrás R, Navarrete J, Bellosta-Batalla M, Martínez-Brotóns C, Martínez-Rubio D. Changes in Salivary Immunoglobulin A, Stress, and Burnout in a Workplace Mindfulness Intervention: A Pilot Study. Int J Environ Res Public Health. 2022;1. doi:[10.3390/ijerph19106226](https://doi.org/10.3390/ijerph19106226)

Mistretta EG, Davis MC, Temkit M, Lorenz C, Darby B, Stonnington CM. Resilience Training for Work-Related Stress among Health Care Workers. J Occup Environ Med. 2018;60: 559–568. doi:[10.1097/JOM.0000000000001285](https://doi.org/10.1097/JOM.0000000000001285)

Moody K, Kramer D, Santizo RO, Magro L, Wyshogrod D, Ambrosio J, et al. Helping the Helpers: Mindfulness Training for Burnout in Pediatric Oncology-A Pilot Program. J Pediatr Oncol Nurs. 2013;30: 275–284. doi:[10.1177/1043454213504497](https://doi.org/10.1177/1043454213504497)

O’Driscoll M, Byrne S, Byrne H, Lambert S, Sahm LJ. An online mindfulness-based intervention for undergraduate pharmacy students: Results of a mixed-methods feasibility study. Currents Pharm Teach Learn. 2019;11: 858–875. doi:[10.1016/j.cptl.2019.05.013](https://doi.org/10.1016/j.cptl.2019.05.013)

O’Driscoll M, Sahm LJ, Byrne H, Lambert S, Byrne S. Impact of a mindfulness-based intervention on undergraduate pharmacy students’ stress and distress: Quantitative results of a mixed-methods study. Currents Pharm Teach Learn. 2019;11: 876–887. doi:[10.1016/j.cptl.2019.05.014](https://doi.org/10.1016/j.cptl.2019.05.014)

Pérez V, Menéndez-Crispín EJ, Sarabia-Cobo C, de Lorena P, Fernández-Rodríguez A, González-Vaca J. Mindfulness-Based Intervention for the Reduction of Compassion Fatigue and Burnout in Nurse Caregivers of Institutionalized Older Persons with Dementia: A Randomized Controlled Trial. Int J Environ Res Public Health. 2022;19. doi:[10.3390/ijerph191811441](https://doi.org/10.3390/ijerph191811441)

Prudenzi A, Graham CD, Flaxman PE, Wilding S, Day F, O’Connor DB. A workplace Acceptance and Commitment Therapy (ACT) intervention for improving healthcare staff psychological distress: A randomised controlled trial. PLoS ONE. 2022;17. doi:[10.1371/journal.pone.0266357](https://doi.org/10.1371/journal.pone.0266357)

Purdie DR, Federman M, Chin A, Winston D, Bursch B, Olmstead R, et al. Hybrid Delivery of Mindfulness Meditation and Perceived Stress in Pediatric Resident Physicians: A Randomized Clinical Trial of In-Person and Digital Mindfulness Meditation. J Clin Psychol Med Settings. 2023;30: 425–434. doi:[10.1007/s10880-022-09896-3](https://doi.org/10.1007/s10880-022-09896-3)

Roeser RW, Schonert-Reichl KA, Jha A, Cullen M, Wallace L, Wilensky R, et al. Mindfulness training and reductions in teacher stress and burnout: Results from two randomized, waitlist-control field trials. Journal of Educational Psychology. 2013;105: 787–804. doi:[10.1037/a0032093](https://doi.org/10.1037/a0032093)

Sairanen E, Lappalainen R, Lappalainen P, Hiltunen A. Mediators of change in online acceptance and commitment therapy for psychological symptoms of parents of children with chronic conditions: An investigation of change processes. Journal of Contextual Behavioral Science. 2020;15: 123–130. doi:[10.1016/j.jcbs.2019.11.010](https://doi.org/10.1016/j.jcbs.2019.11.010)

Schroeder DA, Stephens E, Colgan D, Hunsinger M, Rubin D, Christopher MS. A Brief Mindfulness-Based Intervention for Primary Care Physicians: A Pilot Randomized Controlled Trial. Am J Lifestyle Med. 2018;12: 83–91. doi:[10.1177/1559827616629121](https://doi.org/10.1177/1559827616629121)

Seidel LW, Dane FC, Carter KF. Brief Mindfulness Practice Course for Healthcare Providers. J Nurs Adm. 2021;51: 395–400. doi:[10.1097/NNA.0000000000001035](https://doi.org/10.1097/NNA.0000000000001035)

Singh NN, Lancioni GE, Medvedev ON, Hwang Y-S, Myers RE, Townshend K. Using mindfulness to improve quality of life in caregivers of individuals with intellectual disabilities and autism spectrum disorder. Int J. 2020;66: 370–380. doi:[10.1080/20473869.2020.1827211](https://doi.org/10.1080/20473869.2020.1827211)

Singh NN, Lancioni GE, Medvedev ON, Myers RE, Chan J, McPherson CL, et al. Comparative Effectiveness of Caregiver Training in Mindfulness-Based Positive Behavior Support (MBPBS) and Positive Behavior Support (PBS) in a Randomized Controlled Trial. Mindfulness (N Y). 2020;11: 99–111. doi:[10.1007/s12671-018-0895-2](https://doi.org/10.1007/s12671-018-0895-2)

Smith JL, Allen JW, Haack CI, Wehrmeyer KL, Alden KG, Lund MB, et al. Impact of App-Delivered Mindfulness Meditation on Functional Connectivity, Mental Health, and Sleep Disturbances Among Physician Assistant Students: Randomized, Wait-list Controlled Pilot Study. JMIR Form Res. 2021;5: e24208. doi:[10.2196/24208](https://doi.org/10.2196/24208)

Strub L, Tarquinio C. Effets de la Mindfulness-Based Cognitive Therapy (MBCT) sur le stress et les symptômes associés dans un contexte industriel : une étude-pilote contrôlée et randomisée. smq. 2013;38: 207–225. doi:[10.7202/1019193ar](https://doi.org/10.7202/1019193ar)

Taylor H, Cavanagh K, Field AP, Strauss C. Health Care Workers’ Need for Headspace: Findings From a Multisite Definitive Randomized Controlled Trial of an Unguided Digital Mindfulness-Based Self-help App to Reduce Healthcare Worker Stress. JMIR Mhealth Uhealth. 2022;10: e31744. doi:[10.2196/31744](https://doi.org/10.2196/31744)

Verweij H, van Ravesteijn H, van Hooff MLM, Lagro-Janssen ALM, Speckens AEM. Mindfulness-Based Stress Reduction for Residents: A Randomized Controlled Trial. J Gen Intern Med. 2018;33: 429–436. doi:[10.1007/s11606-017-4249-x](https://doi.org/10.1007/s11606-017-4249-x)

Watanabe N, Horikoshi M, Shinmei I, Oe Y, Narisawa T, Kumachi M, et al. Brief mindfulness-based stress management program for a better mental state in working populations - Happy Nurse Project: A randomized controlled trial. J Affective Disord. 2019;251: 186–194. doi:[10.1016/j.jad.2019.03.067](https://doi.org/10.1016/j.jad.2019.03.067)

Xu H, Eley R, Kynoch K, Tuckett A. Effects of mobile mindfulness on emergency department work stress: A randomised controlled trial. EMA Emerg Med Australas. 2022;34: 176–185. doi:[10.1111/1742-6723.13836](https://doi.org/10.1111/1742-6723.13836)

Zheng Y, Gu X, Jiang M, Zeng X. How might mindfulness-based interventions reduce job burnout? Testing a potential self-regulation model with a randomized controlled trial. Mindfulness. 2022;13: 1907–1922. doi:[10.1007/s12671-022-01927-2](https://doi.org/10.1007/s12671-022-01927-2)
